# Improving male involvement in antenatal care in low and middle-income countries to prevent mother to child transmission of HIV: A realist review

**DOI:** 10.1371/journal.pone.0240087

**Published:** 2020-10-15

**Authors:** Jacinta Clark, Linda Sweet, Simangaliso Nyoni, Paul R. Ward

**Affiliations:** 1 College of Medicine and Public Health, Flinders University, Adelaide, Australia; 2 School of Nursing and Midwifery, Deakin University and Western Health Partnership, Burwood, Australia; 3 College of Nursing and Health Sciences, Flinders University, Adelaide, Australia; University of Cape Town, SOUTH AFRICA

## Abstract

**Background:**

Childhood Human Immunodeficiency Virus (HIV) infection occurs almost exclusively via mother to child transmission (MTCT) during pregnancy, birth, or through breastfeeding. Recent studies have shown that male involvement (MI) in antenatal care (ANC) and HIV testing, including couples voluntary counselling and testing (CVCT), increases the likelihood that women will adhere to prevention advice and comply with HIV treatment if required during their pregnancy; hence reducing the rates of MTCT of HIV. This realist review investigates how, why, when, and for whom MI in ANC works best to provide contextual advice on how MI in ANC can be best used for prevention of mother to child transmission (PMTCT) of HIV.

**Methods:**

A realist review of existing evidence was conducted. Realist review seeks to explain how and why an intervention works, or does not work, in a given context. This was completed through the five stages of realist synthesis; Eliciting the program theory, search strategy, study selection criteria, data extraction, and data analysis and synthesis. Findings are presented as context-mechanism-outcome (CMO) configurations outlining the mechanisms that work in given contexts to give an outcome.

**Results:**

Three CMO configurations were developed. These describe that 1) Couples in monogamous relationships have higher levels of trust, commitment and security leading to increased uptake of PMTCT programs together; 2) ANC spaces that make ‘male friendly’ adaptions promote normalisation of MI in PMTCT and are more welcoming, leading to increased willingness of male partners to participate in ANC; and 3) couples and communities with higher health literacy encourage increased informed decision making, ownership, and responsibility and thus increased participation in PMTCT of HIV.

**Conclusions:**

The CMOs developed in this review give contextual advice on how one might improve ANC services to increase MI and help reduce MTCT of HIV. We propose that MI in ANC works best where couples are monogamous and trusting, where ANC spaces actively promote being a ‘male friendly space’ and where there are high levels of community education programs around MTCT.

## Introduction

In 2019, approximately 150 000 children (<15 years old) globally became newly infected with human immunodeficiency virus (HIV) [1]. While this number may be overshadowed by the 1.5 million new infections that occurred in adults in the same year [1, 2], it holds significance in that these infections effectively represent the global rates of mother to child transmission (MTCT) of HIV [3]. New HIV infection in children occurs almost exclusively via vertical transmission [3]; that is HIV is transmitted to a child from their mother during pregnancy, childbirth, or by breast feeding [4]. An estimated 1.3 million women living with HIV globally become pregnant every year [4, 5]. Without intervention, the risk that a HIV infected mother will transfer the virus to her child during the perinatal and breastfeeding periods ranges from 5–45% [4–6]. However, with appropriate intervention this risk can be reduced to less than 2% [6, 7].

**Fig 1:**
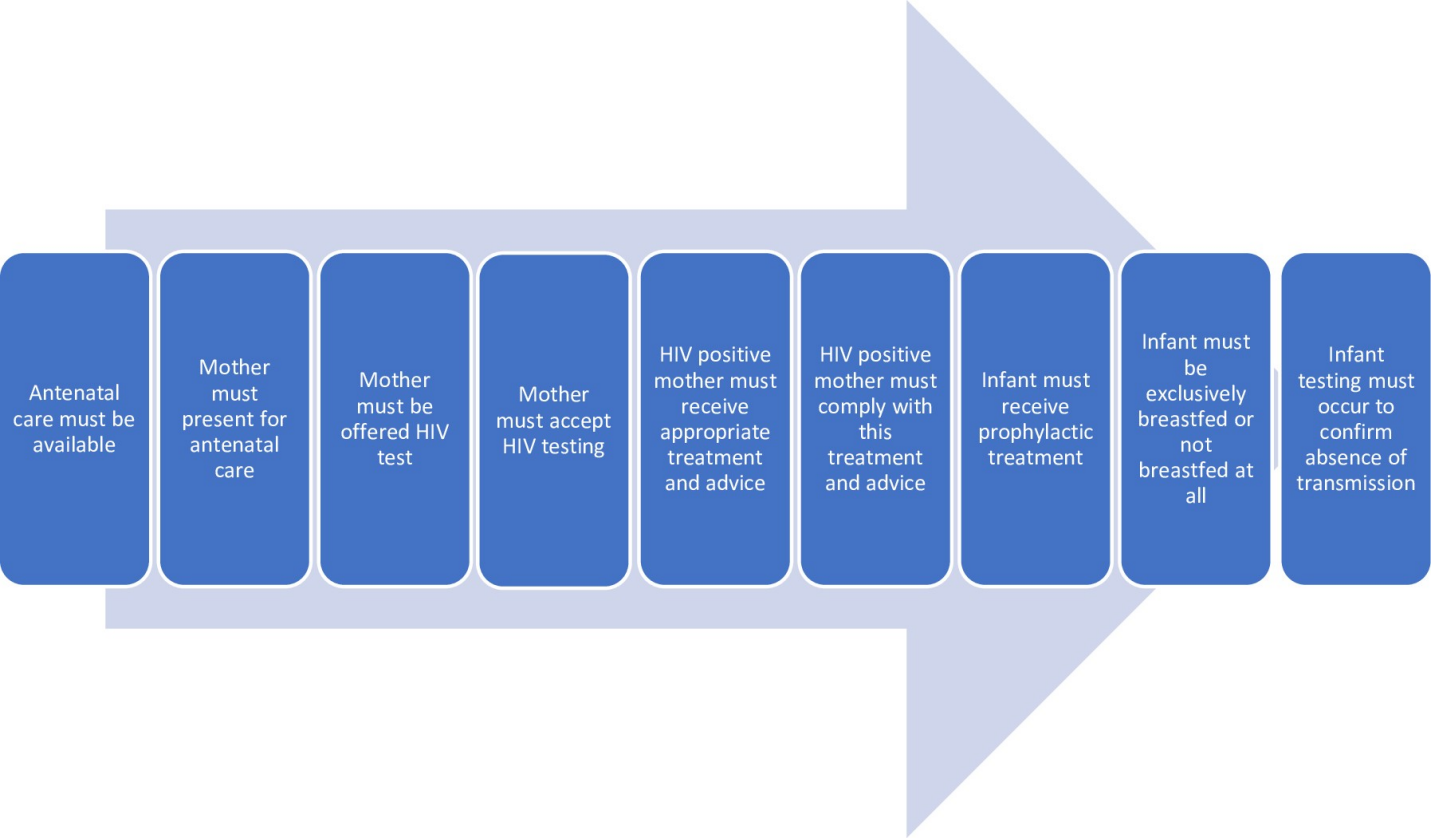
Steps to prevent mother to child transmission of HIV (figure adapted from PMTCT cascade [8]).

The reduction in transmission risk and subsequent prevention of mother to child transmission (PMTCT) is dependent on the pregnant mother being successfully navigated through a series of healthcare steps [3]. These are identified in Fig 1. Each of these steps carries with it numerous barriers which have the potential for loss to follow up of the pregnant women and their infant [3].

PMTCT programs have had a significant impact on rates of vertical transmission. The 150 000 new childhood infections in 2019 is a considerable drop from the 290 000 new infections in 2010 [9, 10]. Despite such improvements, we know there remain significant gaps in achieving best practice and that the main burden of this is focused in low and middle income countries [9, 11]. In 2019 only 85% pregnant women with HIV were provided with antiretroviral therapy (ART) to reduce the risk of MTCT of HIV [1, 2] leaving 25% of HIV positive pregnant women receiving no protection against MTCT [9]. In addition, within the 85% of pregnant women commencing ART, issues with compliance are high [1, 5, 9] thus the risk of MTCT in these women remains.

Historically antenatal care (ANC) and PMTCT programs have focused solely on pregnant woman [12–22], ignoring the reality that these women are not necessarily solely responsible for their decisions, and are not always able to act freely [12–14, 17, 21–28]. In the past decade it has been recognised that several influences affect the behaviours of these women and that many of these relate to their male partners [9, 12–14, 17, 21–27]. In contexts and cultures where patriarchal gender norms dominate, it is likely that the choices being made around sexual practices, contraception and health care are not being made by the woman alone but rather that these decisions are being strongly influenced by her male partner [9, 12–14, 17, 21–29]. However, because PMTCT programs have in the past been so woman-centric [12–22], the men making decisions possibly lack education, and could make ill-informed choices about the health of their partner and unborn child [9, 29].

It has been shown that male involvement (MI) in ANC and PMTCT can increase adherence to PMTCT strategies, and thus has positive effects on rates of MTCT and infant survival [9, 28, 29], however, the success of implementing programs aiming to improve MI seems to vary in different contexts [14]. This demonstrates that this is a complex issue strongly influenced by human decisions and actions. Health professionals know how to prevent HIV transmission, but what we continue to be challenged by are the social and contextual factors to enable effective prevention, particularly for MTCT. While we know MI in PMTCT works as an intervention in some contexts, we do not know which contexts encourage or discourage MI and why, neither do we have a good model of how MI in PMTCT can be implemented most effectively.

### The methodology–Why realist review?

Complex social interventions, such as those relating to MI in PMTCT of HIV, act within complex social systems and their success is based on the decisions made by those individuals being targeted [30]. As such, any choice a given individual makes is dependent on the context in which they are making the decision. It is therefore reasonable to assume that given a different context an individual may come to a different conclusion. It is for this reason that we can expect social interventions delivered in different contexts to yield different outcomes [31]. Traditional forms of research review, such as systematic review, do not necessarily address this reality [30].

In evaluating social interventions, traditional forms of review are likely to give an incomplete picture or uncertain results as they do not take into account the effect of differing contexts [30]. Realist review, on the other hand, is particularly suited to investigating the complex and multifaceted nature of social interventions [31]. While traditional review methods seek to determine whether an intervention is effective or not, realist reviews seek to explain why an intervention may or may not work, for which people, and in what circumstances [32], providing an explanation of *how* it may work rather than just a judgement of whether or not it works [31]. This *how* is presented as a ‘CMO’ configurations which describe the causal relationships being investigated. They describe that in a given context (C), specific mechanisms (M) will activate an anticipated outcome (O) [32]. While a realist review does not give hard and fast answers about the effectiveness of an intervention, it does help develop a deeper and more practical understanding of an intervention so that it may be delivered in the most effective way [30].

A realist review investigating MI in antenatal care, and voluntary counselling and testing for HIV in pregnancy has not been undertaken, and thus this review aims to identify mechanisms that result in the desired program outcomes of increased adherence to PMTCT strategies to inform how MI in PMTCT can be best implemented.

## Methods

### Five stages of realist review

#### Stage 1—Eliciting the program theory

Interventions are theory incarnate [32]; they say that if we deliver these services or this intervention in this way, we will get this outcome [30, 33]. And like all theories, they are open to being supported or refuted. In realist review, the program theory is an articulation of how a given intervention is expected to work and acts as the hypothesis that is being tested. By developing a clear understanding of how a program is expected to work, we give ourselves the best opportunity to compare what is expected to what is actually happening [33]. Hence, realist review begins with development of a program theory to be refined throughout the research [30]. Realist program theories are presented as ‘CMO’ configurations which describe the causal relationships being investigated [32].

To build the initial program theory we conducted an initial ‘scoping review’ of the literature’ [33]. This process involved informally exploring the literature, including both peer-reviewed and grey literature, to establish an initial understanding of the theory of how MI is expected to improve uptake of PMTCT interventions for pregnant women in low and middle-income countries. Numerous sources were used in this process, including reports from international organisations such as UNAIDS and World Health Organisation, existing published research around barriers to the uptake of PMTCT interventions by pregnant women, as well as research around MI in PMTCT programs. Academic literature was sourced through informal searches of Ovid MEDLINE, Cochrane Database of Systematic Reviews and Google scholar using simple combinations of search terms ‘male involvement’, ‘hiv’, ‘antenatal care’, and ‘PMTCT’. This broad reading set the foundation to build a preliminary program theory. The preliminary program theory is presented as a series of context, mechanism, outcome (CMO) configurations to be tested throughout the review (see Table 1). These were developed by theorising reasons that a woman may not progress through each of the steps required to prevent MTCT (Fig 1). Each of these potential missteps was translated into a CMO theory.

**Table 1:** Preliminary program theory.

Context	Mechanism	Outcome
**“Positive Relationships”**	High level of perceived mutual **trust** in relationship	Pregnant woman invites male partner to ANC
**Mutual primary partner,**		
**Monogamous couples,**	Increased intimacy in relationship, level of commitment to relationship	Male partner accepts invitation
**High level of security in relationship,**		Couple consents to testing together
	Couple feel **safe** to participate in testing together	
**Situations of domestic violence**	Woman’s perceives she is **unsafe**	Woman does not invite husband to ANC
	**Fear** of negative repercussions with positive test	
**Situations of infidelity**	Unfaithful partner **fears** repercussions of positive test (divorce, abandonment, decreased quality of relationship)	Woman does not invite male partner
		Male partner refuses to attend
**PMTCT strategies targeted at women/mothers**	Male partners feel **unwelcome, unrepresented, unwanted, excluded, disengaged**	Male partner does not attend ANC for PMTCT
**ANC considered “women’s business”**		
**Male partners formally invited into ANC space**	Male partners feel **welcomed, invited, included**	Male partners attend ANC with pregnant woman
• **Formal letters**		
**ANC set up as “male friendly” space**	Male partners feel sense of **belonging, welcome, invited into space**	Male partners attend ANC with pregnant woman
**Community Engagement around male involvement in PMTCT**	Male partner has **awareness** of role of men in PMTCT & Sense of **responsibility**	Male partner agrees to participate in PMTCT
**Previous exposure to PMTCT**	Reduction in **perception** that PMTCT is a women’s issue	
**Increased community/individual health literacy**		
**CVCT offered in non-ANC spaces, spaces where male partners already attend and feel welcome, E.g. Churches, community-based events**	**Perception** of being CVCT being about partners, families, rather than about ANC and women’s business	Male partners agree to participate in CVCT
	**Normalisation** of Male involvement in PMTCT	
**Male partner educated about PMTCT**	**Learning/growing understanding** about PMTCT and consequences of MTCT	Male partner uses decision making power/influence to aid in PMTCT
**Male partner and pregnant woman are counselled and tested together in CVCT, receive results together facilitating communication**	**Teamwork**	Increased uptake and compliance of PMTCT strategies.
	**Shared values, shared view of importance** of PMTCT, **equal motivation**	
**Community with high social stigma around HIV**	**Fear** around participating	Decreased participation
	**Fear** around knowing status	
**Low health literacy in community/of individual**	**Impaired decision-making ability**	Reduced uptake and participating
	**Impaired risk perception**	
**Male partners play role of providers, need to take time away from work to participate in ANC/PMTCT**	**Prioritising** of earning income over attending ANC	Reduced male participation in ANC
	**Delegation** of tasks, mother delegated task of ANC	
	**Fear** of judgement (ANC is for women)	
	**Fear** of stigmatization for requesting time off of work to get a HIV test	

#### Stage 2—Search strategy

Following development of the initial program theory, the next step was to locate relevant evidence to inform the CMO configuration review. Following the lead of Rycroft-Malone et al. [31] and groups that have completed rapid realist reviews [34], we deviated from pure realist methodology [30] and began a formal search strategy with an electronic database search. This formal search differs from the initial scoping searches in that the scoping search is effectively a process of broad reading to learn about the intervention and context while the formal search is a reproducible and unbiased collection of sources with which you can test your program theory [30]. To ensure an inclusive search, a medical librarian was consulted to improve keyword search strategies and database identification (see S1 Appendix for search terms and strategy). Our initial search was concluded on the 29^th^ of May 2017. As we were aware that our formal search would likely be our only significant gathering of data, we chose to make it as inclusive as possible by choosing a broad selection of databases [30]. This formal search included the databases Ovid MEDLINE, Embase, CINAHL, Cochrane Database of Systematic Reviews, Cochrane Central Register of Controlled Trials, Scopus, Web of Science and ProQuest. Due to a delay in publication, the search was repeated on 12/06/2020 to ensure the most recent data was also included prior to publishing (see S2 Appendix for the search terms and strategy of the repeated search).

#### Stage 3—Study selection criteria

In realist review the traditional hierarchical approach to evidence is rejected because the rich picture you hope to produce requires input from many different forms and sources of evidence [30]. For example, in realist review a randomised control trial is not considered a more reliable source of evidence than a cross sectional study as we aren’t looking for good proof that an intervention works, we are looking for how the intervention seemed to be working in that context and how individuals were responding to it. As one can imagine, qualitative papers with interviews and quotes are particularly valuable in this process whereas in traditional review methodology they would be considered less useful than a randomised control trial. Studies used in a realist review should be diverse, and judgements on the value of a piece of research should not be strictly based on study design but rather on how well it informs the program theory [32]. In selecting literature for this review, we assessed both relevance and rigour. Decisions around relevance of an article were made based on whether it was related to the topic of interest and whether it could contribute to theory building, while our assessment of rigour evaluated whether a particular piece of work was credible and trustworthy [33]. It is important to understand that while a study would not be excluded based on the type of study, it was removed if it was of poor quality [30].

The process of appraisal and selection of studies to be included began with initial screening of title and abstract. The inclusion criteria for this initial screen was as follows

1. Study is published in English with full text available
2. Research focused on low or middle-income country/ies
3. Addresses MI in ANC and/or PMTCT

While these were treated as strict criteria, in the initial stages we tended towards inclusion when the title and abstract appeared to be relevant to ensure good evidence was not missed. Due to the overwhelming number of results from the initial search, we focused on articles published from 2010, with the proviso that if we could return to the pre-2010 results if required (this ended up not being necessary as there was sufficient good quality evidence published after 2010 to reach a point of ‘saturation’ as described by Pawson et al. [30]). Full text of the remaining articles were assessed for rigour using a series of standardised critical appraisal tools from the Joanna Briggs Institute [35] and The Critical Appraisals Skills Programme [36]. These tools provided an objective checklist of criteria for each different type of study design that papers could be marked against. To ensure a manageable number of articles during our data extraction process we strictly removed any articles that did not meet a high-quality standard determined as a score greater than 80% on the appropriate critical appraisal tool.

#### Stage 4—Data extraction

After removing all articles that did not meet the rigour standard, the remaining 38 articles were read in full thoroughly and iteratively to find evidence that related to the initial program theories. The software program NVivo 11 was used to manage the data. A hybrid deductive and inductive thematic analysis was conducted. Articles were searched for evidence that proved or disproved the initial program theory and to identify any new possibilities, and all potentially relevant data was coded into NVivo. This process was undertaken by the lead researcher and to avoid bias was reviewed by a second member of the research team, any and all disagreements were discussed with the team as a whole to be rectified. Following review of all articles the coding structure was reviewed and themes relating to contexts, mechanisms and outcomes were drawn out and sorted.

#### Stage 5—Data analysis and synthesis

The goal in synthesis is to use the data extracted to refine the initial program theory [31]. Realist analysis of data collected requires a combination of not just deductive and inductive reasoning, but also the more abstract abductive and retroductive reasoning [37, 38]. That is, the researcher does not just look for data that proves of disproves their initial theory. They must acknowledge all data available and instead of proving or disproving a theory, they use the data to alter the theory to come to a most likely description of what may be occurring. The data collected in stage 4 was compared to the initial program theory and then used to alter the initial program theory to produce a refined program theory of how context effects the mechanisms that work and result in desired outcomes of MI in PMTCT programs.

## Results

### Search results

**Fig 2:**
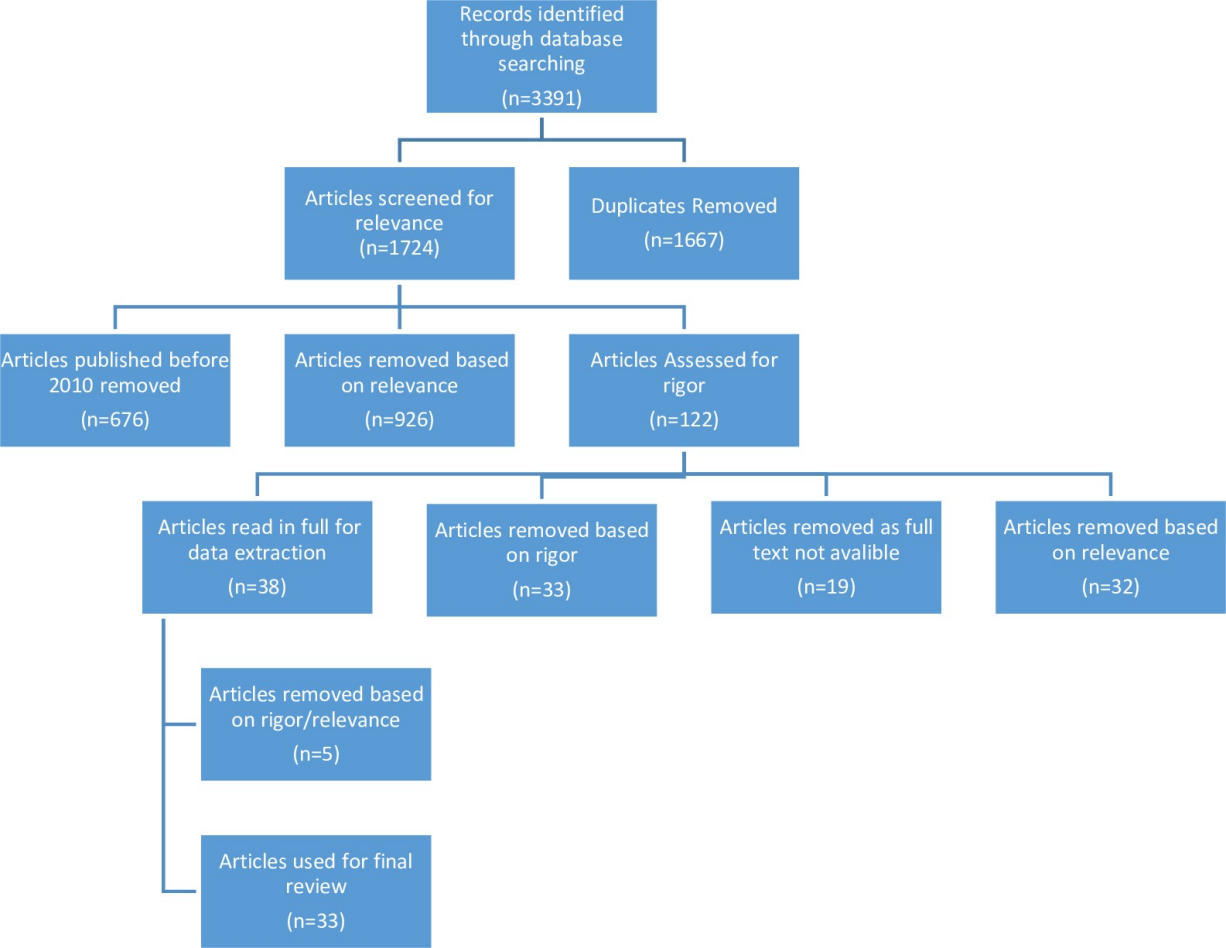
Search results as at 29/05/2017.

The initial search returned 3391 results, 1724 once duplicates were removed. Databases gave varying numbers of results, between 2 and 1201. Following the initial title and abstract screening process, 926 articles were removed as they did not meet the inclusion criteria, and a further 676 articles published between 1998 and 2009 were removed due to publication date; due to the sufficient amount of evidence published post 2010. Thereafter, 122 articles were assessed for rigour, with 33 articles removed for poor rigour, 19 articles were removed as full text was not available, and 32 articles were removed based on not meeting the inclusion criteria. Of the 38 articles read in full for data extraction, a further 5 articles were removed based on relevance or rigour, leaving 33 articles for the final review process (see Fig 2).

**Fig 3:**
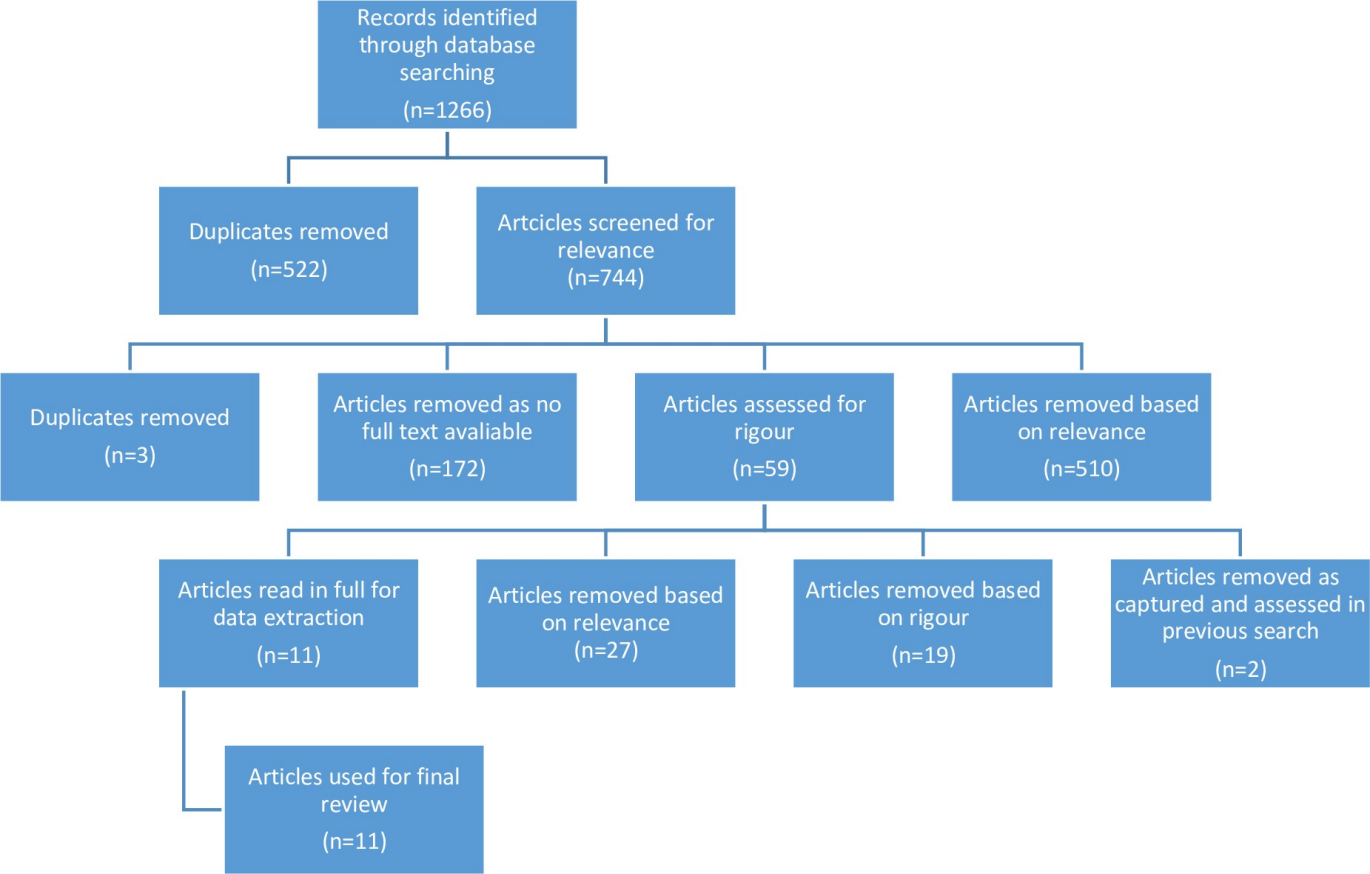
Search results as at 12/06/20.

The repeated search in 2020 returned 1266 results, 744 once duplicates were removed. Databases gave varying numbers of results, between 21 and 458. Following the initial title and abstract screening process, 510 articles were removed as they did not meet the inclusion criteria, a further 3 duplicates were removed at this stage as well as 172 papers that did not have full text available online. The remaining 59 articles were assessed for rigour, with 19 articles being removed for poor rigour, and a further 27 articles being removed based on not meeting the inclusion criteria, a further 2 articles were removed at this stage as they were captured in the previous search conducted in 2017. After rigour assessment 11 articles remained to be read in full for data extraction and inclusion in the review (see Fig 3). The combination of both searches provided 44 articles to be included in the final review process.

### Included studies

Table 2 identifies basic characteristics of study setting and type of the included studies. Of the 44 articles included, 24 were qualitative studies [13, 14, 18–23, 25, 27, 28, 39–51], 10 were cross sectional studies [26, 51–59], 3 were cohort studies [24, 60, 61], 3 were systematic reviews [12, 16, 17], 3 were randomised control trials [62–64], and 1 used mixed methods [15].

**Table 2:** Document characteristics.

Author	Year	Reference Number	Article Title	Country	Study Design
**Aborigo, R. A. et al.**	2018	[28]	Male involvement in maternal health: perspectives of opinion leaders	Ghana	Qualitative
**Adelekan, A. L. et al.**	2014	[18]	Married Men Perceptions and Barriers to Participation in the Prevention of Mother-to-Child HIV Transmission Care in Osogbo, Nigeria	Nigeria	Qualitative
**Aluisio, A. R. et al.**	2016	[61]	Male Partner Participation in Antenatal Clinic Services is Associated With Improved HIV-Free Survival Among Infants in Nairobi, Kenya: A Prospective Cohort Study	Kenya	Cohort Study
**Audet, C. M. et al.**	2016	[26]	Engagement of Men in Antenatal Care Services: Increased HIV Testing and Treatment Uptake in a Community Participatory Action Program in Mozambique	Mozambique	Cross Sectional
**Audet, C. M. et al.**	2016	[46]	Barriers to Male Involvement in Antenatal Care in Rural Mozambique	Mozambique	Qualitative
**Auvinen, J. et al.**	2014	[19]	Midwives' perspectives on male participation in PMTCT of HIV and how they can support it in Lusaka, Zambia	Zambia	Qualitative
**Byamugisha, R. et al.**	2010	[52]	Determinants of male involvement in the prevention of mother-to-child transmission of HIV programme in Eastern Uganda: a cross-sectional survey	Uganda	Cross sectional
**Davis, J. et al.**	2018	[47]	Expectant fathers' participation in antenatal care services in Papua New Guinea: a qualitative inquiry	Papua New Guinea	Qualitative
**Ditekemena, J. et al.**	2012	[16]	Determinants of male involvement in maternal and child health services in sub-Saharan Africa: a review	“Sub Saharan Africa”	Systematic review
**Duff, P. et al.**	2012	[42]	Married men's perceptions of barriers for HIV- positive pregnant women accessing highly active antiretroviral therapy in rural Uganda	Uganda	Qualitative
**Elias, M. et al.**	2017	[56]	Male partner involvement in the prevention of mother to child transmission of HIV infection in Mwanza Region, Tanzania	Tanzania	Cross sectional
**Falnes, E. F. et al.**	2011	[13]	"It is her responsibility": partner involvement in prevention of mother to child transmission of HIV programmes, northern Tanzania	Tanzania	Qualitative
**Galle, A. et al.**	2019	[48]	Policymaker, health provider and community perspectives on male involvement during pregnancy in southern Mozambique: a qualitative study	Mozambique	Qualitative
**Ganle, J.K & Dery, I**	2015	[22]	'What men don’t know can hurt women’s health: a qualitative study of the barriers to and opportunities for men’s involvement in maternal healthcare in Ghana	Ghana	Qualitative
**Gill, M. M. et al.**	2017	[49]	"The co-authors of pregnancy": leveraging men's sense of responsibility and other factors for male involvement in antenatal services in Kinshasa, DRC	Democratic Republic of the Congo	Qualitative
**Haile, F. & Brhan, Y.**	2014	[55]	Male partner involvements in PMTCT: a cross sectional study, Mekelle, Northern Ethiopia	Ethiopia	Cross sectional
**Kalembo, F. W et al.**	2013	[24]	Association between Male Partner Involvement and the Uptake of Prevention of Mother-to-Child Transmission of HIV (PMTCT) Interventions in Mwanza District, Malawi: A Retrospective Cohort Study	Malawi	Cohort study
**Ladur, A. N. et al.**	2015	[20]	Perceptions of Community Members and Healthcare Workers on Male Involvement in Prevention of Mother-To-Child Transmission Services in Khayelitsha, Cape Town, South Africa	South Africa	Qualitative
**Larsson, Elin C. et al.**	2010	[43]	Mistrust in marriage-Reasons why men do not accept couple HIV testing during antenatal care- a qualitative study in eastern Uganda	Uganda	Qualitative
**Maman, S. et al.**	2011	[40]	Defining male support during and after pregnancy from the perspective of HIV-positive and HIV-negative women in Durban, South Africa	South Africa	Qualitative
**Manjate Cuco, R. M. et al.**	2015	[12]	Male partners' involvement in prevention of mother-to-child HIV transmission in sub-Saharan Africa: A systematic review	Uganda, Tanzania, Kenya, Zambia, South Africa, Cameroon, Malawi, Ivory Coast, Democratic Republic of Congo, and Rwanda	Systematic Review
**Matseke, M. G., et al.**	2017	[57]	Factors associated with male partner involvement in programs for the prevention of mother-to-child transmission of HIV in rural South Africa	South Africa	Cross Sectional
**Mohlala, B. K. et al.**	2011	[62]	The forgotten half of the equation: randomized controlled trial of a male invitation to attend couple voluntary counselling and testing	South Africa	Randomised Control Trial
**Morfaw, F. et al.**	2013	[17]	Male involvement in prevention programs of mother to child transmission of HIV: a systematic review to identify barriers and facilitators	Kenya, Uganda, Tanzania, Cote d’Ivoire, Cameroon, Malawi, Democratic Republic of Congo, South Africa, India, Cambodia & France	Systematic review
**Musheke, M. et al.**	2013	[39]	Couple experiences of provider-initiated couple HIV testing in an antenatal clinic in Lusaka, Zambia: lessons for policy and practice	Zambia	Qualitative
**Natoli, L. et al.**	2012	[45]	Promoting safer sexual practices among expectant fathers in the Lao People's Democratic Republic	Lao	Qualitative
**Nyondo, A. L et al.**	2013	[23]	Assessment of strategies for male involvement in the prevention of mother-to-child transmission of HIV services in Blantyre, Malawi	Malawi	Qualitative
**Nyondo, A. L. et al.**	2014	[21]	Stakeholders' perceptions on factors influencing male involvement in prevention of mother to child transmission of HIV services in Blantyre, Malawi	Malawi	Qualitative
**Nyondo, A. L. et al.**	2014	[27]	Exploring the relevance of male involvement in the prevention of mother to child transmission of HIV services in Blantyre, Malawi	Malawi	Qualitative
**Orne-Gliemann, J. et al.**	2010	[15]	Couple-oriented prenatal HIV counselling for HIV primary prevention: an acceptability study	Cameroon, Dominican Republic, Georgia and India.	Mixed—Qualitative and Quantitative
**Orne-Gliemann, J. et al.**	2013	[64]	Increasing HIV testing among male partners	Cameroon, Dominican Republic, Georgia, India	Randomised Control Trial
**Osoti, A. O. et al.**	2015	[53]	Home-based HIV testing for men preferred over clinic-based testing by pregnant women and their male partners, a nested cross-sectional study	Kenya	Cross Sectional
**Osoti, A. O. et al.**	2014	[63]	Home visits during pregnancy enhance male partner HIV counselling and testing in Kenya: a randomized clinical trial	Kenya	Randomised Control Trial
**Oyugi, E. et al.**	2017	[58]	Male partner involvement in efforts to eliminate mother-to-child transmission of HIV in Kisumu County, Western Kenya, 2015	Kenya	Cross sectional
**Rogers, A. J., et al.**	2016	[50]	Couple interdependence impacts HIV-related health behaviours among pregnant couples in southwestern Kenya: a qualitative analysis	Kenya	Qualitative
**Sileo, K. M. et al.**	2016	[14]	"That would be good, but most men are afraid of coming to the clinic": Men and women's perspectives on strategies to increase male involvement in women's reproductive health services in rural Uganda	Uganda	Qualitative
**Theuring, S. et al.**	2010	[44]	Partner involvement in perinatal care and PMTCT services in Mbeya Region, Tanzania: the providers' perspective	Tanzania	Qualitative
**Tweheyo, R. et al.**	2010	[54]	Male partner attendance of skilled antenatal care in peri-urban Gulu district, Northern Uganda	Uganda	Cross sectional
**Villar-Loubet, O. M. et al.**	2013	[41]	HIV disclosure, sexual negotiation and male involvement in prevention-of-mother-to-child-transmission in South Africa	South Africa	Qualitative
**Yeganeh, N. et al.**	2017	[25]	Barriers and facilitators for men to attend prenatal care and obtain HIV voluntary counseling and testing in Brazil	Brazil	Qualitative
**Yeganeh, N. et al.**	2014	[60]	HIV testing of male partners of pregnant women in Porto Alegre, Brazil: a potential strategy for reduction of HIV seroconversion during pregnancy	Brazil	Cohort Study
**Yeganeh, N. et al.**	2017	[51]	Barriers and facilitators for men to attend prenatal care and obtain HIV voluntary counseling and testing in Brazil	Brazil	Qualitative
**Yende, N et al.**	2017	[51]	Acceptability and Preferences among Men and Women for Male Involvement in Antenatal Care	South Africa	Cross sectional
**Zenebe, A. et al.**	2016	[59]	Male Partner's Involvement in HIV Counselling and Testing and Associated Factors among Partners of Pregnant Women in Gondar Town, Northwest Ethiopia	Ethiopia	Cross sectional

Studies were set in a number of countries including (in order of frequency) South Africa [12, 17, 20, 40, 41, 51, 57, 62], Kenya [12, 17, 50, 53, 58, 61, 63], Uganda [12, 14, 17, 42, 43, 52, 54], Malawi [12, 17, 21, 23, 24, 27], Tanzania [12, 13, 17, 44, 56], Cameroon [12, 15, 17, 64], Zambia [12, 19, 39], India [15, 17, 64], Democratic Republic of Congo [12, 17, 49], Mozambique [26, 46, 48], Brazil [25, 51, 60], Ethiopia [55, 59], Dominican Republic [15, 64], Georgia [15, 64], Cote d’Ivoire [12, 17], Ghana [22, 28], Rwanda [12], Cambodia [17], Nigeria [18], Papua New Guinea [47], and Laos [45]. One systematic review used in our review also included data from a group of African migrants in France [17]; this paper was retained in our review as the rest of the data it used came from low and middle income countries.

### Results

The review process allowed for the collection of evidence to refine the initial program theory. During this process 3 key themes were evident allowing us to combine what were many CMO configurations in our initial program theory into 3 refined key CMOs (see Table 3).

**Table 3:** Refined program theory.

	Context	Mechanism	Outcome
**1**	**Nature of relationship**	High levels of perceived mutual **trust** in relationship	Both partners willing to disclose HIV status to the other
	• **Monogamous couples**		
			Pregnant woman invites male partner to ANC
	• **No infidelity**	Increased levels of **intimacy**	
			Male partner accepts invitation
	• **High levels of security in relationship**	Increased levels of **commitment** to relationship	
			Couple consent to testing together/CVCT
	• **No domestic violence**	Couple **feel safe** to participate together	Mutual disclosure
	• **Marriage**	Feel **secure** in relationship	
	• **Stable relationship**		
		Shared **ownership**	
**2**	**How ‘male friendly’ ANC space is**	Male partners feel **welcome, invited, belonging, included, engaged**	Male partner attends ANC with pregnant partner
	• **Formal invitation to ANC for male partners**		
	• **Space for male partners**		
	• **Hours acceptable to male partners**	**Normalisation** of male involvement in ANC	
	• **Male staff members**	**Perception** of ANC being about partners and families	
	• **Health activities for men/incentives**		
	• **‘family clinic’ rather than ‘women’s clinic’**		
	• **Community based ANC activities**		
**3**	**Increased engagement through increased health literacy**	**Learning** and **growing understanding** about PMTCT and consequences of MTCT	Pregnant partner invites male partner to ANC
	• **Community based education**		
			Male partner accepts invitation to ANC
	• **Male leaders promoting MI in PMTCT**	Individuals have **awareness** of role of men in PMTCT	
			Couple participate in PMTCT program
		Reduction in **perception** that PMTCT is a women’s issue	
	• **Previous experience with PMTCT**		
		**Normalisation** of male involvement in PMTCT	
		**Informed** decision making	
		Appropriate **perception** of risk	
		**Ownership** and **responsibility** around own health	

#### CMO1—The nature of a couple’s relationship influences whether they are likely to participate in PMTCT programs together or not

From the review it is clear that MI in PMTCT works best for couples in monogamous [14, 15, 50, 56, 57], trusting [13], and safe [13, 16, 23, 39, 40] relationships. The degree to which these aspects are perceived by the couple directly relates to the likelihood of being willing to attend ANC with a partner.

There were several factors that seemed to influence how willing an individual would be to disclose their status to their partner, male or female. The more committed they were in a relationship, the more likely they were to participate in couples voluntary counselling and testing (CVCT) as a part of ANC.

“Women, who perceived their relationship as supportive, loving and trusting would disclose without fear of rejection or abandonment” [41] pg 264

Non-monogamous couples were not as willing as monogamous couples [54] to attend for CVCT.

Being married increased a couple’s likelihood of participating in CVCT [12, 50, 60], although infidelity was repeatedly mentioned as a barrier to MI in any ANC or subsequent CVCT [14, 15, 52]. If either partner had participated in extramarital sexual relationships, the likelihood they would agree to participate in CVCT as a part of ANC was reduced [48, 50]. This included women refusing a test if they knew their partner had been unfaithful as they did not want to bring a positive test home and risk taking the blame [46]. In contrast, men who had been faithful to their pregnant partners reported willingness to participate in a HIV test if it were offered to them [15].

“If you engaged in sex with other men and suspect yourself to be HIV positive, you wouldn’t like to come with your partner in the clinic because it might cause misunderstandings in the home.”– 33-year-old pregnant woman attending ANC alone [14] pg 1557

“For those women who have a good, open and honest relationship with their husband it’s fine to bring their husband. For those who are married to the … euhm the typical macho man, it’s difficult, they are not open, there is no trust. They are scared to invite their husband, to bring her husband … They are scared to be accused of HIV infection.” Female provider, age group 30–40, Provider FGD [48] pg 7.

High levels of trust and stability in a relationship were associated with increased likelihood of participating in CVCT [39, 46, 48, 59, 65]. Distrustful or unstable relationships were associated with decreased willingness to participate for both men and women [17]. While disclosure has been suggested to not significantly increase rates of domestic violence or relationship breakdown [62, 64], the fear of these is a very real barrier to MI in PMTCT. If a woman has any fear that disclosure will lead to domestic violence or abandonment it is very likely that she will not disclose [16, 17, 21, 39, 55–57, 62]. It is evident that the more secure a couple feel in their relationship the more willing they are to be tested for HIV together [17, 39].

“As to why some men did go for couple HIV testing, the men we interviewed speculated that these were men who had a good relationship with their wives, and had marriages marked by mutual love, trust, and understanding.” [43] pg 4

In relation to CMO1, we theorise that relationship characteristics that lead to high levels of mutual trust, intimacy, commitment, safety, security and ownership encourages willingness to disclose ones’ HIV status and therefore increases a couple’s willingness to participate in CVCT as a part of ANC.

#### CMO2—How ‘male friendly’ the ANC space is influences how likely male partners are to attend

Men tend to prefer not to attend ANC services that are dominated by women and increase their involvement when male friendly adjustments are made to services. Comments from male participants from a range of studies reaffirmed their current dissatisfaction with ANC and offered suggestions on how to improve men’s engagement with ANC [12, 17, 21, 25, 45, 65].

Across multiple studies men and women alike refer to ANC as women’s business [12–22, 28, 47]. This idea has been so strongly engrained, it has led to ANC being labelled as “unmanly” in many cultures [15–17, 22, 25, 28, 46–48, 65]. There is strong social pressures around this in countries where patriarchal norms dominate with men risking ridicule and judgement for attending a “women’s clinic” [28, 46–48].

“Even the songs we always sing at the antenatal clinic, we always mention the woman, not a man, so the men feel ashamed to be together with their wives, they feel that they are not part of it”. Key informant [21] pg7

“Men who accompanied their wives to the clinic were called ‘kana-kodona’ (women’s rivals) or ‘bakana’ which means ‘man-woman’; suggesting that the man exhibits female tendencies.” [28] pg6

“They come fetch you up saying "Let´s go for a walk. Why are you being commanded by your wife?” (Man, 20, bike taxi driver, Gonhane)” [46] pg 7

“Providing support to a pregnant partner, including accompaniment to an ANC appointment, meant that men had to endure the heckling and mocking of their friends. Providing physical or emotional support to a pregnant partner implied male weakness.” [46] pg 6

ANC being considered a women’s space is a significant barrier to MI in PMTCT programs [23]. In the female dominated ANC clinic, those men who do attend feel out of place, unwelcome and unnecessary [12, 17, 45, 51].

“Attending the antenatal clinic was seen as “unmanly” to the extent that men feared being socially stigmatized if they accompanied their wives to the antenatal clinic” [13] pg 9

Men tend to prefer receiving VCT in ‘friendlier’ spaces outside of the ANC setting [16, 17] and are more likely than women to request home-based testing over ANC-based testing [53]. As well as men not feeling as though they belong in the ANC space, it is often reported that they are not treated as though they are welcome in the space [22].

“many of the men implied that the prenatal care clinic was not inviting” [25] pg 11

Throughout multiple studies, men reported that staff were unfriendly to them [15, 19, 28, 47, 64], that they were not included in the appointment [14, 19, 22, 25, 41, 43, 45, 48], even that they were asked to leave the clinic [16, 19, 20, 22, 28, 44, 52], all of which discouraged the men from returning to the ANC clinic.

“Sometimes, the nurses are always so harsh and they don’t want to see a man (husband) inside the maternity ward.” [22] pg 8

The review identified that male participation increased at clinics that made “male friendly” adjustments to their usual practice [13, 45, 51]. These included providing incentives for male partners to attend, formally inviting male partners to attend, and employing male staff members. Incentives included preferential treatment for women who attended with their male partner to reduce wait times [21, 27, 39, 47, 49], providing health checks for the male partners [12, 21], providing parenting advice for the male partners [12, 20, 51], even providing gifts for couples who attended together [21]. The act of formally inviting male partners into the ANC space appeared to have a significant effect on how willing the male partner was to attend ANC [12, 17, 23, 47, 49, 51, 54, 62, 65].

“Involve us in the registration, antenatal check-up, getting drugs and all the processes to make us feel that we are also important people in the clinic.” (Man accompanying partner, age 32) [14] pg 1559

“If the doctor sent me an invitation, I cannot refuse to answer. I'll be going to know my HIV status.” (Male FGD participant) [49] pg 5

It seemed the formality of the process made men feel invited and welcome [25], made them feel the process was legitimate [43] and reassured them that they had a role to play in the ANC space [27].

“Few of the participants added a condition that their presence must be needed at the clinic before they could accompany their wife.” [18] pg 4

“Health care providers involved with the couple counselling activities at the Vientiane MCHH said that most of the men who are invited do come along to these special sessions, suggesting that men are interested to receive information if they feel welcome.” [45] pg 306

In addition to men being more likely to accept a formal invitation from the clinic, women preferred having a formal invitation to legitimise their request of their partner’s attendance [13, 14, 43, 46, 51]. It is not uncommon for women to feel intimidated in asking their partners to attend ANC, and so a formal invitation from the clinic also reduces this anxiety for the pregnant woman [13, 14, 43, 51].

“I think, for a man to believe that the wife’s message is true; there should be a written notification. … Not much will be said on the paper, the letter ought to be stamped with a hospital stamp. When the man sees the stamp he will know that he is wanted.” [23] pg 5

The incorporation of male staff and volunteers attempts to balance gender in a female dominated space [12, 14, 26, 39, 46, 47]. This strategy seems to normalise the presence of men in the ANC setting [39] and the longer the male staff/volunteers are involved, the more acceptable MI seems to become for the community [26]. This normalisation is further reinforced as men continue to attend the clinic [20, 66].

In relation to CMO2, men in low and middle-income countries respond to being invited into ANC and are more willing to participate in CVCT and PMTCT as a part of ANC when the space is more ‘male friendly’.

#### CMO3—The health literacy of a couple and their community influences how likely they are to participate in PMTCT programs

General awareness and knowledge of PMTCT increases how likely an individual is to willingly participate in PMTCT strategies including CVCT. It was clear from the evidence that health literacy around HIV and PMTCT was generally low [12–15, 17, 18, 21, 25, 41, 42, 49, 54], which acted as a barrier to men participating in ANC and PMTCT programs.

Men’s incorrect beliefs around contraceptive and condom use [13, 14, 41] and particularly beliefs around the pregnant woman’s HIV status automatically being the same as her husbands [12, 13, 15, 17, 21, 25, 41], fed the belief that the male partner did not need to attend ANC or participate in PMTCT strategies. Men were less likely to have received any targeted education about HIV or prevention strategies and were subsequently more likely to hold these incorrect beliefs compared to women [25, 42]. Comparatively, men who had participated in ANC previously or had previous experience with HIV testing or treatment had greater understanding of the process and its importance and were as a result more willing to participate in PMTCT programs again [17, 20, 49, 52, 54, 56, 58, 59, 61, 63, 64].

“The findings … show that expectant fathers need and want more information so that they can better protect the health of their partners and babies during and after pregnancy, and that they are willing to attend antenatal care when invited” [45] pg 307

An individual’s level of general education was also an indicator of how willing they were to participate in ANC and PMTCT programs. Higher levels of education of the male or female partner was associated with higher levels of participation in PMTCT [12, 16, 24, 49, 51, 52, 57, 58]. Being young and living in urbanised areas were factors associated with receiving higher levels of education, and subsequently having a greater awareness and appreciation of why PMTCT programs were important [22].

“The problem is that there are many men … especially in rural areas who have no formal education. Such men do not always understand the risks involved in getting pregnant and giving birth” (Male Participant, FGD) [22] pg 10

As well as increasing an individual’s knowledge of PMTCT being important, when thinking about male partners it is also important to consider how that knowledge can be increased. For male partners, strategies to increase health literacy around PMTCT that were led by men were particularly successful. These included male community leaders publicly advocating for PMTCT [21, 23, 28, 48, 49], and male employees and volunteers in the ANC clinic acting as educators and peer supports [21, 22, 26, 46]. Men also suggested that they would appreciate hearing from other men who had experience with HIV as role models [43].

Evidence for CMO3 suggest that having greater health literacy around HIV and PMTCT strategies and, for men in particular, having respected sources to learn from, increases an individual’s willingness to participate in PMTCT programs.

## Discussion

This is the first realist review to explore the context-mechanism-outcome configurations around MI in PMTCT of HIV in low and middle-income countries. What our findings identify is that a PMTCT of HIV service delivered to monogamous and trusting couples, in a male friendly space, in a community with high health literacy around PMTCT and HIV is likely to have high attendance, retention and compliance, encouraging low rates of MTCT of HIV. However, what we know is that this ideal situation is rarely, if ever, the case. PMTCT of HIV service providers face numerous challenges working in the field. Services are regularly underfunded and understaffed [16, 20, 21, 25] making it difficult for services to also play the role of community advocates or to even incorporate MI as it could near double their workload. In light of this, we appreciate that real world application of these recommendations will be far more difficult than it was for us to write them, however, we hope that in compiling this information we can offer guidance on how to better implement MI in PMTCT where it is possible.

Our first CMO configuration identified that the nature of a couple’s relationship influences how likely they were to participate in PMTCT programs together. Couples who were monogamous and had high levels of security and trust in their relationship were the most likely couples to first present for ANC together and go to forth with CVCT. Because ANC is so targeted to women, the majority of PMTCT programs wanting to incorporate MI rely on the pregnant woman inviting her male partner to attend ANC with her [13]. For this to occur, the pregnant woman must be willing to disclose a potentially positive result to her male partner [41]. If she has reservations about her partner knowing her status, she will not volunteer to be tested with him. Similarly, for the male partner to accept an invitation to participate in ANC, he must be willing to disclose a potentially positive result to his pregnant partner [25]. We determined that in the context of a “positive” relationship, i.e. a monogamous, safe and trusting relationship, couples were more willing to participate in CVCT together. Of the three CMO configurations, this is the most difficult for health professionals to influence, as they do not have control over the nature of a couple’s relationships, as much of this is influenced by patriarchal and cultural norms. What they can do is understand how the mechanism of a "positive" relationship works, to assist identifying which couples are most likely to accept CVCT and to appreciate that some couples will not be willing for CVCT, and ensure we identify alternate pathways for them.

A number of clinical trials looking at the influence of MI on PMTCT of HIV regularly allowed the women to self-select which arm of the trial they would participate in, the intervention group (MI group) or the control group (no MI group), making the trials non-randomised. It is clear that this was often allowed to ensure that women did not disengage with ANC entirely, however we removed a number of these studies early in our review based on the bias this created. In these studies, MI groups were found to have higher levels of compliance and lower rates of transmission, however, what these studies could have actually been telling us is that women who felt comfortable inviting their partner to attend ANC with them were more likely to comply, regardless of whether their partner was involved or not. If women who are in safe and supportive environment feel more secure involving their male partner in ANC and subsequent CVCT, one could argue that a woman who feels safe inviting her partner would also have felt safe disclosing her status to her partner whether he was involved in testing or not.

Our second CMO configuration described how welcome men feel in the female dominated ANC space. Men who felt welcome and needed at the clinic were far more likely to attend for ANC with their partner. ANC, across both the developed and developing worlds, is regularly considered within the domain of women’s health [12–22]. Extending from this, we know that ANC spaces are often considered female spaces; it is not uncommon for antenatal clinics to be referred to as women’s clinics and staff are often predominately female [12]. While this is the case across the world, it is exaggerated in the developing world as patriarchal norms are far more accepted and gender roles are more culturally relevant and strictly enforced [12–14, 17, 19, 21–27, 39, 43].

There were a number of suggested ways to alter context to make it more male friendly. Perhaps the most significant of these, with the best evidence, was offering formal written invitation to the male partners officially inviting them into the space. This has been shown repeatedly to increase attendance of male partners to ANC clinics [12, 17]. It was also evident that the more routine MI was perceived to be, the more accepted it was as a practice [13, 17, 21]. This normalisation of MI in ANC, by making it the expected standard of care, made ANC a more male friendly space [17].

There are several small changes clinics can make to make their space more male friendly. Careful choice of language is important, as ANC clinics being referred to as “women’s clinics” reinforces the female-ness of the space [45]. Being careful to not remove ownership for women, clinics can shift to family-based language, for example prevention of parent to child transmission (PPTCTT) rather than PMTCT [21], family clinic rather than women’s clinic [12], and potentially delivering ANC is spaces that are already gender neutral like outpatient clinics [21] could all be helpful. Clinics can take steps towards reorientation of services to serve both sexes, providing parenting advice for both mother and father. For some clinics, the first step may even be simply allowing men to attend appointments with their partners. Our review suggests that even the smallest of steps to improve how welcome male partners feel within the clinic will improve their willingness to attend ANC and subsequent CVCT.

Our third CMO was perhaps the most expected—pregnant women and male partners who have a good understanding of why PMTCT strategies are important are more likely to attend for CVCT and adhere to treatment. It is well known that HIV is associated with a level of stigma which has a significant effect on how willing individuals are to engage with testing and treatment [13, 14, 17, 19, 25, 67]. We opted to not investigate this barrier in a CMO configuration as it has been well documented and is a universal issue when talking about HIV rather than being explicitly related to MI in PMTCT. However, relating to our third CMO configuration, we know areas with high health literacy and easy access to health information have reduced levels of stigma [67]. A suggested mechanism of increasing men’s engagement in particular was to have the education in this area being delivered by other men or ‘male champions’, as male partners in these overwhelmingly patriarchal societies tend to respond more strongly to male leadership [21–23, 26, 28]. Community engagement with the issue is key to ensuring that individuals understand the need to present for ANC, agree to screening and adhere to treatment. With each new generation, there is the possibility that cultural norms alter, including those related to gender roles/politics. Widespread community engagement can encourage a norm where men are expected to participate in ANC [21].

Completing this realist review has allowed us to tease out how and why MI in PMTCT works in some contexts and not in others. Unlike other more traditional forms of review, realist review does not control for real life events and instead allows the researcher to investigate how real life stimulates the different mechanisms that work to give certain outcomes in different contexts [31]. It provided us with a flexible methodology that allowed inquisitive investigation of the mechanisms that work to give both positive and negative outcomes in different contexts [31].

While we do refer to our review as a realist review, we acknowledge that we have followed the lead of other research groups [31, 34] and deviated from pure realist methodology as outlined by Pawson et al. [30]. Pure realist methodology promotes an iterative data collection process with repeated searches outside the subject area to better understand the mechanisms that work (e.g. it may be possible to see the mechanisms we identified working in other subject areas associated with similar contexts and investigation around this may have increased our understanding of them) [30, 32]. Our initial scoping of the literature was very open and iterative, however we switched to a more systematic strategy once we began our formal search. We acknowledge that this may have limited the depth of our understanding, however stand by our choice as the most efficient way to complete this review.

Realist review, unlike more traditional forms of review, relies on the researcher’s interpretation of the data collected to form conclusions. Our team made our best effort to reduce bias in the results by having the data extraction as well as the data analysis and synthesis stages reviewed by a second team member. As mentioned in the methods, any inconsistencies were brought to the team as a whole to be discussed and rectified.

Realist methodology as a system for review is in itself limited in the territory that you can cover; interventions have multiple stages and it may not be possible for one review to cover every stage [30]. This was true of our review, and thus we focused on the involvement of male partners in ANC, aware that this meant there were a number of issues that would not be addressed in our results [30]. Our realist approach also limits our results in that they may not be generalisable to all pregnant women and male partners, for example couples in high income countries or in countries where gender politics are more equal may not respond in the way we have predicted [31]. We are also not able to give hard and fast recommendations for improving MI in PMTCT [30]. Rather our realist approach enables us to give contextual advice around what may work in certain areas [30].

## Conclusion

Our review is the first to use available evidence to develop a program theory that attempts to explain how and why MI in PMTCT programs works (or does not work) in specific contexts. The evidence demonstrated that couples in monogamous, safe and trusting relationships were best suited to CVCT, that male partners are more willing to attend ANC in male friendly spaces, and that couples and communities with high health literacy around PMTCT of HIV were most likely to engage with PMTCT programs. It is our hope that this review can offer some contextual advice in how MI in PMTCT of HIV programs might be best implemented in low and middle-income countries to increase HIV testing, increase treatment compliance and reduce MTCT and childhood infection with HIV.

## Supporting information

S1 Appendix Search terms and results as at 29/05/17. (DOCX)

S2 Appendix Search terms and results as at12/06/20. (DOCX)

## Abbreviations

MI: Male Involvement
VCT: voluntary counselling and testing
CVCT: couple’s voluntary counselling and testing
MTCT: Mother to child transmission
PMTCT: Prevention of Mother to Child Transmission
PPTCT: Prevention of Parent to Child Transmission
ANC: Antenatal care
HIV: Human Immunodeficiency Virus
CMO: Context-Mechanism-Outcome
