# Supplementary material for: Improving male involvement in antenatal care in low and middle-income countries to prevent mother to child transmission of HIV: A realist review
Source: PLoS One. 2020 Oct 15;15(10):e0240087. doi: 10.1371/journal.pone.0240087 (PMC7561142; doi:10.1371/journal.pone.0240087)
Supplement: S1 Appendix — (DOCX) [file pone.0240087.s001.docx]

# S1 Appendix – Search Terms and Results as at 29/05/17

## S1 Appendix details the search strategy undertaken up to 29/05/20. Databases utilised were Ovid MEDLINE, Embase, CINAHL, Cochrane Database of Systematic Reviews, Cochrane Central Register of Controlled Trials, Scopus, Web of Science and ProQuest.

| **Database** | **Citations retrieved (n)** |
| --- | --- |
| Ovid MEDLINE(R) Epub Ahead of Print, In-Process & Other Non-Indexed Citations, Ovid MEDLINE(R) Daily, Ovid MEDLINE and Versions(R) | 1164 |
| Embase (Ovid), 1974 to 2017 May 26 | 1201 |
| CINAHL (EBSCOhost) | 107 |
| Cochrane Database of Systematic Reviews: Issue 5 of 12, May 2017 | 2 |
| Cochrane Central Register of Controlled Trials: Issue 4 of 12, April 2017 | 30 |
| Scopus | 231 |
| Web of Science | 212 |
| ProQuest (Health & Medicine; Social Sciences subsets) | 309 |
| Total before duplicates removed | 3256 |
| Total after duplicates removed | 1724 |

## Ovid MEDLINE(R) Epub Ahead of Print, In-Process & Other Non-Indexed Citations, Ovid MEDLINE(R) Daily, Ovid MEDLINE and Versions(R)

| **#** | **Searches** | **Results** |
| --- | --- | --- |
| 1 | Pregnancy/ OR Pregnant women/ OR Prenatal care/ | 819255 |
| 2 | (Pregnan* OR prenatal* OR antenatal* OR reproductive health).tw,kw. | 533486 |
| 3 | 1 OR 2 | 952133 |
| 4 | Pregnancy Complications, Infectious/ OR Infectious Disease Transmission, Vertical/ | 42212 |
| 5 | ((("Mother* to child*" OR "maternal to child" OR vertical*) adj2 transmi*) OR PMTCT OR MTCT).tw,kw. | 11090 |
| 6 | 4 OR 5 | 47305 |
| 7 | HIV seropositivity/ OR HIV-1/ OR HIV infections/ | 218389 |
| 8 | (HIV* OR human immunodeficiency virus OR PLWHA OR PLWHIV OR PLHIV).tw,kw. | 299224 |
| 9 | 7 OR 8 | 325577 |
| 10 | (3 OR 6) and 9 | 24217 |
| 11 | Men/ OR Male/ OR Fathers/ OR Family Characteristics/ OR Spouses/ OR Sex Factors/ OR Sexual partners/ OR Interpersonal relations/ | 7757706 |
| 12 | ((Male* OR men* OR partner* OR husband* OR spous* OR couple*) adj4 (involve* OR attend* OR participat* OR return OR accompan* OR engagement OR object* OR opposition* OR support*)).tw,kw. | 53323 |
| 13 | gender*.ti. | 36978 |
| 14 | 11 OR 12 OR 13 | 7785796 |
| 15 | Counseling/ OR Directive Counseling/ OR Patient education as topic/ OR Behavior therapy/ OR Cognitive Therapy/ OR Voluntary Programs/ OR Health Education/ OR Health Promotion/ OR Family Planning Services/ | 286825 |
| 16 | (counsel* OR antenatal care OR ANC OR (Couple* and ("testing and counsel?ing" OR "Counsel?ing and testing" OR HTC OR VCT)) OR CHTC OR CVCT).tw,kw. | 99584 |
| 17 | 15 OR 16 | 359289 |
| 18 | Developing countries/ | 70080 |
| 19 | (LMIC OR ((Developing OR Majority OR Less developed OR low resource* OR disadvantaged OR resource limited OR poor OR low* income* OR "low and middle income*") adj3 (countr* OR region* OR nation? OR area* OR econom*))).tw,kw. | 85981 |
| 20 | Caribbean region/ OR west indies/ OR cuba/ OR dominica/ OR dominican republic/ OR grenada/ OR haiti/ OR jamaica/ OR saint lucia/ OR "saint vincent and the grenadines"/ OR americas/ OR central america/ OR belize/ OR costa rica/ OR el salvador/ OR guatemala/ OR honduras/ OR nicaragua/ OR panama/ OR "gulf of mexico"/ OR latin america/ OR mexico/ OR south america/ OR argentina/ OR bolivia/ OR brazil/ OR colombia/ OR ecuador/ OR guyana/ OR paraguay/ OR peru/ OR suriname/ OR venezuela/ OR asia/ OR asia, central/ OR kazakhstan/ OR kyrgyzstan/ OR tajikistan/ OR turkmenistan/ OR uzbekistan/ OR asia, northern/ OR russia/ OR siberia/ OR asia, southeastern/ OR cambodia/ OR east timor/ OR indonesia/ OR laos/ OR malaysia/ OR myanmar/ OR philippines/ OR thailand/ OR vietnam/ OR asia, western/ OR bangladesh/ OR bhutan/ OR india/ OR afghanistan/ OR iraq/ OR jordan/ OR lebanon/ OR syria/ OR turkey/ OR yemen/ OR nepal/ OR pakistan/ OR sri lanka/ OR china/ OR "democratic people's republic of korea"/ OR mongolia/ OR europe, eastern/ OR albania/ OR bosnia-herzegovina/ OR bulgaria/ OR kosovo/ OR "macedonia (republic)"/ OR moldova/ OR montenegro/ OR "republic of belarus"/ OR romania/ OR serbia/ OR ukraine/ OR fiji/ OR papua new guinea/ OR vanuatu/ OR micronesia/ OR guam/ OR palau/ OR samoa/ OR american samoa/ OR tonga/ OR africa/ OR africa, northern/ OR algeria/ OR egypt/ OR libya/ OR morocco/ OR tunisia/ OR "africa south of the sahara"/ OR africa, central/ OR cameroon/ OR central african republic/ OR chad/ OR congo/ OR "democratic republic of the congo"/ OR equatorial guinea/ OR gabon/ OR africa, eastern/ OR burundi/ OR djibouti/ OR eritrea/ OR ethiopia/ OR kenya/ OR rwanda/ OR somalia/ OR sudan/ OR tanzania/ OR uganda/ OR africa, southern/ OR angola/ OR botswana/ OR lesotho/ OR malawi/ OR mozambique/ OR namibia/ OR south africa/ OR swaziland/ OR zambia/ OR zimbabwe/ OR africa, western/ OR benin/ OR burkina faso/ OR cape verde/ OR cote d'ivoire/ OR gambia/ OR ghana/ OR guinea/ OR guinea-bissau/ OR liberia/ OR mali/ OR mauritania/ OR niger/ OR nigeria/ OR senegal/ OR sierra leone/ OR togo/ | 867585 |
| 21 | (caribbean* OR west indi* OR cuba* OR dominica* OR grenada* OR haiti* OR jamaica* OR saint lucia* OR "saint vincent and the grenadines" OR central america* OR belize* OR costa rica* OR el salvador* OR guatemala* OR hondura* OR nicaragua* OR panama* OR latin america* OR mexic* OR south america* OR argentin* OR bolivia* OR brazil* OR colombia* OR ecuador* OR guyana* OR paraguay* OR peru* OR suriname* OR venezuela* OR asia* OR kazakhstan* OR kyrgyzstan* OR tajikistan* OR turkmenistan* OR uzbekistan* OR russia* OR siberia* OR cambodia* OR east timor* OR indonesia* OR lao* OR malaysia* OR myanmar* OR philippin* OR thai* OR vietnam* OR bangladesh* OR bhutan* OR india* OR afghanistan* OR iraq* OR jordan* OR leban* OR syria* OR turkey* OR turkish* OR yemen* OR nepal* OR pakistan* OR sri lanka* OR china* OR chinese OR korea* OR mongolia* OR albania* OR bosnia-herzegovina* OR bulgaria* OR kosovo* OR macedonia* OR moldov* OR montenegro* OR belarus* OR romania* OR serbia* OR ukraine* OR fiji* OR papua* OR vanuatu* OR micronesia* OR guam* OR palau* OR samoa* OR tonga* OR africa* OR algeria* OR egypt* OR libya* OR morocc* OR tunisia* OR cameroon* OR central african republic* OR chad* OR cong* OR equatorial guinea* OR gabon* OR burundi* OR djibouti* OR eritrea* OR ethiopia* OR kenya* OR rwanda* OR somalia* OR sudan* OR tanzania* OR uganda* OR angola* OR botswana* OR lesotho* OR malawi* OR mozambique* OR namibia* OR south africa* OR swaziland* OR zambia* OR zimbabwe* OR benin* OR burkina faso* OR cape verde* OR cote d'ivoire* OR gambia* OR ghana* OR guinea* OR guinea-bissau* OR liberia* OR mali* OR mauritania* OR niger* OR senegal* OR sierra leone* OR togo*).tw,kw. | 2220465 |
| 22 | 18 OR 19 OR 20 OR 21 | 2522232 |
| 23 | 10 and 14 and 17 and 22 | 1206 |
| 24 | limit 23 to english language | 1164 |

## Embase 1974 to 2017 May 26

| **#** | **Searches** | **Results** |
| --- | --- | --- |
| 1 | adolescent pregnancy/ or pregnancy/ | 613080 |
| 2 | pregnant woman/ | 54516 |
| 3 | prenatal care/ | 32720 |
| 4 | reproductive health/ | 12813 |
| 5 | (Pregnan* or prenatal* or antenatal* or reproductive health).tw,kw. | 635023 |
| 6 | or/1-5 | 919510 |
| 7 | pregnancy complication/ | 70732 |
| 8 | vertical transmission/ | 12434 |
| 9 | ((("Mother* to child*" or "maternal to child" or vertical*) adj2 transmi*) or PMTCT or MTCT).tw,kw. | 13591 |
| 10 | or/7-9 | 89459 |
| 11 | human immunodeficiency virus infection/ or human immunodeficiency virus 1 infection/ | 254400 |
| 12 | (HIV* or human immunodeficiency virus or PLWHA or PLWHIV or PLHIV).tw,kw. | 358210 |
| 13 | or/11-12 | 409207 |
| 14 | (6 or 10) and 13 | 28480 |
| 15 | male/ | 7253177 |
| 16 | father/ | 16460 |
| 17 | spouse/ or husband/ | 13176 |
| 18 | married person/ or married man/ | 5295 |
| 19 | sex difference/ | 337373 |
| 20 | ((Male* or men* or partner* or husband* or spous* or couple*) adj4 (involve* or attend* or participat* or return or accompan* or engagement or object* or opposition* or support*)).tw,kw. | 68435 |
| 21 | gender*.ti. | 45480 |
| 22 | or/15-21 | 7375850 |
| 23 | counseling/ or directive counseling/ or e-counseling/ or family counseling/ or patient counseling/ or patient guidance/ | 98984 |
| 24 | patient education/ | 99080 |
| 25 | behavior therapy/ | 40606 |
| 26 | cognitive therapy/ | 41620 |
| 27 | couple therapy/ | 108 |
| 28 | health education/ | 88303 |
| 29 | health promotion/ | 82727 |
| 30 | family planning/ | 35696 |
| 31 | (counsel* or antenatal care or ANC or (Couple* and ("testing and counsel?ing" or "Counsel?ing and testing" or HTC or VCT)) or CHTC or CVCT).tw,kw. | 134992 |
| 32 | or/23-31 | 508795 |
| 33 | developing country/ | 86518 |
| 34 | (LMIC or ((Developing or Majority or Less developed or low resource* or disadvantaged or resource limited or poor or low* income* or "low and middle income*") adj3 (countr* or region* or nation? or area* or econom*))).tw,kw. | 103991 |
| 35 | (caribbean* or west indi* or cuba* or dominica* or grenada* or haiti* or jamaica* or saint lucia* or "saint vincent and the grenadines" or central america* or belize* or costa rica* or el salvador* or guatemala* or hondura* or nicaragua* or panama* or latin america* or mexic* or south america* or argentin* or bolivia* or brazil* or colombia* or ecuador* or guyana* or paraguay* or peru* or suriname* or venezuela* or asia* or kazakhstan* or kyrgyzstan* or tajikistan* or turkmenistan* or uzbekistan* or russia* or siberia* or cambodia* or east timor* or indonesia* or lao* or malaysia* or myanmar* or philippin* or thai* or vietnam* or bangladesh* or bhutan* or india* or afghanistan* or iraq* or jordan* or leban* or syria* or turkey* or turkish* or yemen* or nepal* or pakistan* or sri lanka* or china* or chinese or korea* or mongolia* or albania* or bosnia-herzegovina* or bulgaria* or kosovo* or macedonia* or moldov* or montenegro* or belarus* or romania* or serbia* or ukraine* or fiji* or papua* or vanuatu* or micronesia* or guam* or palau* or samoa* or tonga* or africa* or algeria* or egypt* or libya* or morocc* or tunisia* or cameroon* or central african republic* or chad* or cong* or equatorial guinea* or gabon* or burundi* or djibouti* or eritrea* or ethiopia* or kenya* or rwanda* or somalia* or sudan* or tanzania* or uganda* or angola* or botswana* or lesotho* or malawi* or mozambique* or namibia* or south africa* or swaziland* or zambia* or zimbabwe* or benin* or burkina faso* or cape verde* or cote d'ivoire* or gambia* or ghana* or guinea* or guinea-bissau* or liberia* or mali* or mauritania* or niger* or senegal* or sierra leone* or togo*).tw,kw. | 2822883 |
| 36 | or/33-35 | 2907703 |
| 37 | 14 and 22 and 32 and 36 | 1225 |
| 38 | limit 37 to english language | 1201 |

## Web of Science; N=212

| # 5 | [212](http://apps.webofknowledge.com.ezproxy.flinders.edu.au/summary.do?product=WOS&doc=1&qid=9&SID=Z1rnEo8ePCCN1J3I5vT&search_mode=CombineSearches&update_back2search_link_param=yes) | #3 AND #2 AND #1  **Refined by:** **LANGUAGES:** (ENGLISH)  Indexes=SCI-EXPANDED, SSCI, A&HCI, CPCI-S, CPCI-SSH, ESCI, CCR-EXPANDED, IC Timespan=All years |
| --- | --- | --- |
| # 4 | [213](http://apps.webofknowledge.com.ezproxy.flinders.edu.au/summary.do?product=WOS&doc=1&qid=8&SID=Z1rnEo8ePCCN1J3I5vT&search_mode=CombineSearches&update_back2search_link_param=yes) | #3 AND #2 AND #1  Indexes=SCI-EXPANDED, SSCI, A&HCI, CPCI-S, CPCI-SSH, ESCI, CCR-EXPANDED, IC Timespan=All years |
| # 3 | [22,538](http://apps.webofknowledge.com.ezproxy.flinders.edu.au/summary.do?product=WOS&doc=1&qid=5&SID=Z1rnEo8ePCCN1J3I5vT&search_mode=GeneralSearch&update_back2search_link_param=yes) | **TOPIC:** ((counsel* OR “antenatal care” OR ANC OR (Couple* and ("testing and counseling" OR "testing and counselling" OR "Counseling and testing" OR "Counselling and testing" OR HTC OR VCT)) OR CHTC OR CVCT)) *AND* **TOPIC:** ((LMIC OR ((Developing OR Majority OR “Less developed” OR “low resource*” OR disadvantaged OR “resource limited” OR poor OR “low income*” OR “lower income” OR "low and middle income*") NEAR/2 (countr* OR region* OR nation? OR area* OR econom*)) OR caribbean* OR “west indi*” OR cuba* OR dominica* OR grenada* OR haiti* OR jamaica* OR “saint lucia*” OR "saint vincent and the grenadines" OR “central america*” OR belize* OR “costa rica*” OR “el salvador*” OR guatemala* OR hondura* OR nicaragua* OR panama* OR “latin america*” OR mexic* OR “south america*” OR argentin* OR bolivia* OR brazil* OR colombia* OR ecuador* OR guyana* OR paraguay* OR peru* OR suriname* OR venezuela* OR asia* OR kazakhstan* OR kyrgyzstan* OR tajikistan* OR turkmenistan* OR uzbekistan* OR russia* OR siberia* OR cambodia* OR “east timor*” OR indonesia* OR lao* OR malaysia* OR myanmar* OR philippin* OR thai* OR vietnam* OR bangladesh* OR bhutan* OR india* OR afghanistan* OR iraq* OR jordan* OR leban* OR syria* OR turkey* OR turkish* OR yemen* OR nepal* OR pakistan* OR “sri lanka*” OR china* OR chinese OR korea* OR mongolia* OR albania* OR “bosnia-herzegovina*” OR bulgaria* OR kosovo* OR macedonia* OR moldov* OR montenegro* OR belarus* OR romania* OR serbia* OR ukraine* OR fiji* OR papua* OR vanuatu* OR micronesia* OR guam* OR palau* OR samoa* OR tonga* OR africa* OR algeria* OR egypt* OR libya* OR morocc* OR tunisia* OR cameroon* OR “central african republic*” OR chad* OR cong* OR “equatorial guinea*” OR gabon* OR burundi* OR djibouti* OR eritrea* OR ethiopia* OR kenya* OR rwanda* OR somalia* OR sudan* OR tanzania* OR uganda* OR angola* OR botswana* OR lesotho* OR malawi* OR mozambique* OR namibia* OR “south africa*” OR swaziland* OR zambia* OR zimbabwe* OR benin* OR “burkina faso*” OR “cape verde*” OR “cote d'ivoire*” OR gambia* OR ghana* OR guinea* OR “guinea-bissau*” OR liberia* OR mali* OR mauritania* OR niger* OR senegal* OR “sierra leone*” OR togo*))  Indexes=SCI-EXPANDED, SSCI, A&HCI, CPCI-S, CPCI-SSH, ESCI, CCR-EXPANDED, IC Timespan=All years |
| # 2 | [167,561](http://apps.webofknowledge.com.ezproxy.flinders.edu.au/summary.do?product=WOS&doc=1&qid=4&SID=Z1rnEo8ePCCN1J3I5vT&search_mode=GeneralSearch&update_back2search_link_param=yes) | **TOPIC:** (((Male* OR men* OR partner* OR husband* OR spous* OR couple*) NEAR/3 (involve* OR attend* OR participat* OR return OR accompan* OR engagement OR object* OR opposition* OR support*))) *OR* **TITLE:** (gender*)  Indexes=SCI-EXPANDED, SSCI, A&HCI, CPCI-S, CPCI-SSH, ESCI, CCR-EXPANDED, IC Timespan=All years |
| # 1 | [16,946](http://apps.webofknowledge.com.ezproxy.flinders.edu.au/summary.do?product=WOS&doc=1&qid=1&SID=Z1rnEo8ePCCN1J3I5vT&search_mode=GeneralSearch&update_back2search_link_param=yes) | **TOPIC:** ((Pregnan* OR prenatal* OR “pre-natal*” OR antenatal* OR “ante-natal*” OR “reproductive health” OR (("Mother to child*" OR “mothers to child*” OR "maternal to child" OR vertical*) NEAR/1 transmi*) OR PMTCT OR MTCT) AND (HIV* OR “human immunodeficiency virus” OR PLWHA OR PLWHIV OR PLHIV))  Indexes=SCI-EXPANDED, SSCI, A&HCI, CPCI-S, CPCI-SSH, ESCI, CCR-EXPANDED, IC Timespan=All years |

## Cochrane Database of Systematic Reviews: Issue 5 of 12, May 2017; N=2

(Pregnan* OR prenatal* OR “pre-natal*” OR antenatal* OR “ante-natal*” OR “reproductive health” OR (("Mother to child*" OR “mothers to child*” OR "maternal to child" OR vertical*) NEAR/1 transmi*) OR PMTCT OR MTCT) AND (HIV* OR “human immunodeficiency virus” OR PLWHA OR PLWHIV OR PLHIV) AND (((Male* OR men* OR partner* OR husband* OR spous* OR couple*) NEAR/3 (involve* OR attend* OR participat* OR return OR accompan* OR engagement OR object* OR opposition* OR support*)) OR gender*) AND (counsel* OR “antenatal care” OR ANC OR (Couple* AND ("testing and counseling" OR "testing and counselling" OR "Counseling and testing" OR "Counselling and testing" OR HTC OR VCT)) OR CHTC OR CVCT) AND (LMIC OR ((Developing OR Majority OR “Less developed” OR “low resource*” OR disadvantaged OR “resource limited” OR poor OR “low income*” OR “lower income” OR "low and middle income*") NEAR/2 (countr* OR region* OR nation? OR area* OR econom*)) OR caribbean* OR “west indi*” OR cuba* OR dominica* OR grenada* OR haiti* OR jamaica* OR “saint lucia*” OR "saint vincent and the grenadines" OR “central america*” OR belize* OR “costa rica*” OR “el salvador*” OR guatemala* OR hondura* OR nicaragua* OR panama* OR “latin america*” OR mexic* OR “south america*” OR argentin* OR bolivia* OR brazil* OR colombia* OR ecuador* OR guyana* OR paraguay* OR peru* OR suriname* OR venezuela* OR asia* OR kazakhstan* OR kyrgyzstan* OR tajikistan* OR turkmenistan* OR uzbekistan* OR russia* OR siberia* OR cambodia* OR “east timor*” OR indonesia* OR lao* OR malaysia* OR myanmar* OR philippin* OR thai* OR vietnam* OR bangladesh* OR bhutan* OR india* OR afghanistan* OR iraq* OR jordan* OR leban* OR syria* OR turkey* OR turkish* OR yemen* OR nepal* OR pakistan* OR “sri lanka*” OR china* OR chinese OR korea* OR mongolia* OR albania* OR “bosnia-herzegovina*” OR bulgaria* OR kosovo* OR macedonia* OR moldov* OR montenegro* OR belarus* OR romania* OR serbia* OR ukraine* OR fiji* OR papua* OR vanuatu* OR micronesia* OR guam* OR palau* OR samoa* OR tonga* OR africa* OR algeria* OR egypt* OR libya* OR morocc* OR tunisia* OR cameroon* OR “central african republic*” OR chad* OR cong* OR “equatorial guinea*” OR gabon* OR burundi* OR djibouti* OR eritrea* OR ethiopia* OR kenya* OR rwanda* OR somalia* OR sudan* OR tanzania* OR uganda* OR angola* OR botswana* OR lesotho* OR malawi* OR mozambique* OR namibia* OR “south africa*” OR swaziland* OR zambia* OR zimbabwe* OR benin* OR “burkina faso*” OR “cape verde*” OR “cote d'ivoire*” OR gambia* OR ghana* OR guinea* OR “guinea-bissau*” OR liberia* OR mali* OR mauritania* OR niger* OR senegal* OR “sierra leone*” OR togo*)

## Cochrane Central Register of Controlled Trials: Issue 4 of 12, April 2017; N=30

(Pregnan* OR prenatal* OR “pre-natal*” OR antenatal* OR “ante-natal*” OR “reproductive health” OR (("Mother to child*" OR “mothers to child*” OR "maternal to child" OR vertical*) NEAR/1 transmi*) OR PMTCT OR MTCT) AND (HIV* OR “human immunodeficiency virus” OR PLWHA OR PLWHIV OR PLHIV) AND (((Male* OR men* OR partner* OR husband* OR spous* OR couple*) NEAR/3 (involve* OR attend* OR participat* OR return OR accompan* OR engagement OR object* OR opposition* OR support*)) OR gender*) AND (counsel* OR “antenatal care” OR ANC OR (Couple* AND ("testing and counseling" OR "testing and counselling" OR "Counseling and testing" OR "Counselling and testing" OR HTC OR VCT)) OR CHTC OR CVCT) AND (LMIC OR ((Developing OR Majority OR “Less developed” OR “low resource*” OR disadvantaged OR “resource limited” OR poor OR “low income*” OR “lower income” OR "low and middle income*") NEAR/2 (countr* OR region* OR nation? OR area* OR econom*)) OR caribbean* OR “west indi*” OR cuba* OR dominica* OR grenada* OR haiti* OR jamaica* OR “saint lucia*” OR "saint vincent and the grenadines" OR “central america*” OR belize* OR “costa rica*” OR “el salvador*” OR guatemala* OR hondura* OR nicaragua* OR panama* OR “latin america*” OR mexic* OR “south america*” OR argentin* OR bolivia* OR brazil* OR colombia* OR ecuador* OR guyana* OR paraguay* OR peru* OR suriname* OR venezuela* OR asia* OR kazakhstan* OR kyrgyzstan* OR tajikistan* OR turkmenistan* OR uzbekistan* OR russia* OR siberia* OR cambodia* OR “east timor*” OR indonesia* OR lao* OR malaysia* OR myanmar* OR philippin* OR thai* OR vietnam* OR bangladesh* OR bhutan* OR india* OR afghanistan* OR iraq* OR jordan* OR leban* OR syria* OR turkey* OR turkish* OR yemen* OR nepal* OR pakistan* OR “sri lanka*” OR china* OR chinese OR korea* OR mongolia* OR albania* OR “bosnia-herzegovina*” OR bulgaria* OR kosovo* OR macedonia* OR moldov* OR montenegro* OR belarus* OR romania* OR serbia* OR ukraine* OR fiji* OR papua* OR vanuatu* OR micronesia* OR guam* OR palau* OR samoa* OR tonga* OR africa* OR algeria* OR egypt* OR libya* OR morocc* OR tunisia* OR cameroon* OR “central african republic*” OR chad* OR cong* OR “equatorial guinea*” OR gabon* OR burundi* OR djibouti* OR eritrea* OR ethiopia* OR kenya* OR rwanda* OR somalia* OR sudan* OR tanzania* OR uganda* OR angola* OR botswana* OR lesotho* OR malawi* OR mozambique* OR namibia* OR “south africa*” OR swaziland* OR zambia* OR zimbabwe* OR benin* OR “burkina faso*” OR “cape verde*” OR “cote d'ivoire*” OR gambia* OR ghana* OR guinea* OR “guinea-bissau*” OR liberia* OR mali* OR mauritania* OR niger* OR senegal* OR “sierra leone*” OR togo*)

## Scopus; N=231

(TITLE-ABS-KEY((Pregnan* OR prenatal* OR "pre-natal*" OR antenatal* OR "ante-natal*" OR "reproductive health" OR (("Mother to child*" OR "mothers to child*" OR "maternal to child" OR vertical*) W/1 transmi*) OR PMTCT OR MTCT) AND (HIV* OR "human immunodeficiency virus" OR PLWHA OR PLWHIV OR PLHIV))) AND (TITLE-ABS-KEY(((Male* OR men* OR partner* OR husband* OR spous* OR couple*) W/3 (involve* OR attend* OR participat* OR return OR accompan* OR engagement OR object* OR opposition* OR support*))) OR TITLE(gender*)) AND (TITLE-ABS-KEY((counsel* OR "antenatal care" OR ANC OR (Couple* and ("testing and counsel*ing" OR "Counsel*ing and testing" OR HTC OR VCT)) OR CHTC OR CVCT) AND (LMIC OR ((Developing OR Majority OR "Less developed" OR "low resource*" OR disadvantaged OR "resource limited" OR poor OR "low income*" OR "lower income" OR "low and middle income*") W/2 (countr* OR region* OR nation? OR area* OR econom*)) OR caribbean* OR "west indi*" OR cuba* OR dominica* OR grenada* OR haiti* OR jamaica* OR "saint lucia*" OR "saint vincent and the grenadines" OR "central america*" OR belize* OR "costa rica*" OR "el salvador*" OR guatemala* OR hondura* OR nicaragua* OR panama* OR "latin america*" OR mexic* OR "south america*" OR argentin* OR bolivia* OR brazil* OR colombia* OR ecuador* OR guyana* OR paraguay* OR peru* OR suriname* OR venezuela* OR asia* OR kazakhstan* OR kyrgyzstan* OR tajikistan* OR turkmenistan* OR uzbekistan* OR russia* OR siberia* OR cambodia* OR "east timor*" OR indonesia* OR lao* OR malaysia* OR myanmar* OR philippin* OR thai* OR vietnam* OR bangladesh* OR bhutan* OR india* OR afghanistan* OR iraq* OR jordan* OR leban* OR syria* OR turkey* OR turkish* OR yemen* OR nepal* OR pakistan* OR "sri lanka*" OR china* OR chinese OR korea* OR mongolia* OR albania* OR "bosnia-herzegovina*" OR bulgaria* OR kosovo* OR macedonia* OR moldov* OR montenegro* OR belarus* OR romania* OR serbia* OR ukraine* OR fiji* OR papua* OR vanuatu* OR micronesia* OR guam* OR palau* OR samoa* OR tonga* OR africa* OR algeria* OR egypt* OR libya* OR morocc* OR tunisia* OR cameroon* OR "central african republic*" OR chad* OR cong* OR "equatorial guinea*" OR gabon* OR burundi* OR djibouti* OR eritrea* OR ethiopia* OR kenya* OR rwanda* OR somalia* OR sudan* OR tanzania* OR uganda* OR angola* OR botswana* OR lesotho* OR malawi* OR mozambique* OR namibia* OR "south africa*" OR swaziland* OR zambia* OR zimbabwe* OR benin* OR "burkina faso*" OR "cape verde*" OR "cote d'ivoire*" OR gambia* OR ghana* OR guinea* OR "guinea-bissau*" OR liberia* OR mali* OR mauritania* OR niger* OR senegal* OR "sierra leone*" OR togo*))) AND ( LIMIT-TO ( LANGUAGE,"English" ) )

## ProQuest; N=309

Includes the following databases:

- ERIC
- Health & Medical Collection
- Health Management Database
- India Database
- Middle East and Africa Database
- Nursing & Allied Health Database
- Psychology Database
- Public Health Database
- Social Science Database
- Sociology Database
- ProQuest Dissertations & Theses Global
- ProQuest Social Sciences Premium Collection
- Applied Social Sciences Index and Abstracts (ASSIA)
- International Bibliography of the Social Sciences (IBSS)
- PAIS Index
- Social Science Database
- Sociological Abstracts
- Sociology Database

all((Pregnan* OR prenatal* OR "pre-natal*" OR antenatal* OR "ante-natal*" OR "reproductive health" OR (("Mother to child*" OR "mothers to child*" OR "maternal to child" OR vertical*) NEAR/1 transmi*) OR PMTCT OR MTCT) AND (HIV* OR "human immunodeficiency virus" OR PLWHA OR PLWHIV OR PLHIV) AND (((Male* OR men* OR partner* OR husband* OR spous* OR couple*) NEAR/3 (involve* OR attend* OR participat* OR return OR accompan* OR engagement OR object* OR opposition* OR support*)) OR gender*) AND (counsel* OR "antenatal care" OR ANC OR (Couple* AND ("testing and counseling" OR "testing and counselling" OR "Counseling and testing" OR "Counselling and testing" OR HTC OR VCT)) OR CHTC OR CVCT) AND (LMIC OR ((Developing OR Majority OR "Less developed" OR "low resource*" OR disadvantaged OR "resource limited" OR poor OR "low income*" OR "lower income" OR "low and middle income*") NEAR/2 (countr* OR region* OR nation? OR area* OR econom*)) OR caribbean* OR "west indi*" OR cuba* OR dominica* OR grenada* OR haiti* OR jamaica* OR "saint lucia*" OR "saint vincent and the grenadines" OR "central america*" OR belize* OR "costa rica*" OR "el salvador*" OR guatemala* OR hondura* OR nicaragua* OR panama* OR "latin america*" OR mexic* OR "south america*" OR argentin* OR bolivia* OR brazil* OR colombia* OR ecuador* OR guyana* OR paraguay* OR peru* OR suriname* OR venezuela* OR asia* OR kazakhstan* OR kyrgyzstan* OR tajikistan* OR turkmenistan* OR uzbekistan* OR russia* OR siberia* OR cambodia* OR "east timor*" OR indonesia* OR lao* OR malaysia* OR myanmar* OR philippin* OR thai* OR vietnam* OR bangladesh* OR bhutan* OR india* OR afghanistan* OR iraq* OR jordan* OR leban* OR syria* OR turkey* OR turkish* OR yemen* OR nepal* OR pakistan* OR "sri lanka*" OR china* OR chinese OR korea* OR mongolia* OR albania* OR "bosnia-herzegovina*" OR bulgaria* OR kosovo* OR macedonia* OR moldov* OR montenegro* OR belarus* OR romania* OR serbia* OR ukraine* OR fiji* OR papua* OR vanuatu* OR micronesia* OR guam* OR palau* OR samoa* OR tonga* OR africa* OR algeria* OR egypt* OR libya* OR morocc* OR tunisia* OR cameroon* OR "central african republic*" OR chad* OR cong* OR "equatorial guinea*" OR gabon* OR burundi* OR djibouti* OR eritrea* OR ethiopia* OR kenya* OR rwanda* OR somalia* OR sudan* OR tanzania* OR uganda* OR angola* OR botswana* OR lesotho* OR malawi* OR mozambique* OR namibia* OR "south africa*" OR swaziland* OR zambia* OR zimbabwe* OR benin* OR "burkina faso*" OR "cape verde*" OR "cote d'ivoire*" OR gambia* OR ghana* OR guinea* OR "guinea-bissau*" OR liberia* OR mali* OR mauritania* OR niger* OR senegal* OR "sierra leone*" OR togo*))

## CINAHL (EBSCOhost); N=107

| **#** | **Query** | **Limiters/Expanders** | **Results** |
| --- | --- | --- | --- |
| S1 | (MH "Pregnancy") | Search modes - Boolean/Phrase | 105,620 |
| S2 | (MH "Expectant Mothers") | Search modes - Boolean/Phrase | 2,626 |
| S3 | (MH "Prenatal Care") | Search modes - Boolean/Phrase | 9,159 |
| S4 | (MH "Reproductive Health") | Search modes - Boolean/Phrase | 2,916 |
| S5 | TI ( Pregnan* or prenatal* or or "pre-natal*" OR antenatal* or "ante-natal*" OR "reproductive health" ) OR AB ( Pregnan* or prenatal* or or "pre-natal*" OR antenatal* or "ante-natal*" OR "reproductive health" ) | Search modes - Boolean/Phrase | 61,525 |
| S6 | S1 OR S2 OR S3 OR S4 OR S5 | Search modes - Boolean/Phrase | 125,148 |
| S7 | (MH "Pregnancy Complications, Infectious") | Search modes - Boolean/Phrase | 2,637 |
| S8 | (MH "Disease Transmission, Vertical") | Search modes - Boolean/Phrase | 3,269 |
| S9 | TI ( ((("Mother* to child*" or "mothers to child*" OR "maternal to child" or vertical*) N1 transmi*) or PMTCT or MTCT) ) OR AB ( ((("Mother* to child*" or "mothers to child*" OR "maternal to child" or vertical*) N1 transmi*) or PMTCT or MTCT) ) | Search modes - Boolean/Phrase | 1,175 |
| S10 | S7 OR S8 OR S9 | Search modes - Boolean/Phrase | 5,563 |
| S11 | (MH "Human Immunodeficiency Virus") OR (MH "HIV-1") | Search modes - Boolean/Phrase | 4,370 |
| S12 | (MH "HIV Seropositivity") | Search modes - Boolean/Phrase | 3,214 |
| S13 | TI ( HIV* or "human immunodeficiency virus" or PLWHA or PLWHIV or PLHIV) ) OR AB ( HIV* or "human immunodeficiency virus" or PLWHA or PLWHIV or PLHIV) ) | Search modes - Boolean/Phrase | 50,369 |
| S14 | S11 OR S12 OR S13 | Search modes - Boolean/Phrase | 51,835 |
| S15 | S6 OR S10 | Search modes - Boolean/Phrase | 126,356 |
| S16 | S14 AND S15 | Search modes - Boolean/Phrase | 4,825 |
| S17 | (MH "Men") OR (MH "Married Men") | Search modes - Boolean/Phrase | 2,996 |
| S18 | (MH "Spouses") | Search modes - Boolean/Phrase | 6,047 |
| S19 | (MH "Sexual Partners") | Search modes - Boolean/Phrase | 4,157 |
| S20 | (MH "Sex Factors") | Search modes - Boolean/Phrase | 58,070 |
| S21 | TI ( ((Male* or men* or partner* or husband* or spous* or couple*) N3 (involve* or attend* or participat* or return or accompan* or engagement or object* or opposition* or support*)) ) OR AB ( ((Male* or men* or partner* or husband* or spous* or couple*) N3 (involve* or attend* or participat* or return or accompan* or engagement or object* or opposition* or support*)) ) | Search modes - Boolean/Phrase | 14,083 |
| S22 | TI gender | Search modes - Boolean/Phrase | 10,525 |
| S23 | S17 OR S18 OR S19 OR S20 OR S21 OR S22 | Search modes - Boolean/Phrase | 85,430 |
| S24 | (MH "Couples Counseling") OR (MH "Counseling") | Search modes - Boolean/Phrase | 16,682 |
| S25 | (MH "HIV Education") OR (MH "Patient Education") OR (MH "Health Education") | Search modes - Boolean/Phrase | 60,619 |
| S26 | (MH "Health Promotion") | Search modes - Boolean/Phrase | 35,548 |
| S27 | (MH "Behavior Therapy") OR (MH "Cognitive Therapy") | Search modes - Boolean/Phrase | 13,838 |
| S28 | TI ( ((counsel* OR “antenatal care” OR ANC OR (Couple* and ("testing and counseling" OR "testing and counselling" OR "Counseling and testing" OR "Counselling and testing" OR HTC OR VCT)) OR CHTC OR CVCT)) ) OR AB ( ((counsel* OR “antenatal care” OR ANC OR (Couple* and ("testing and counseling" OR "testing and counselling" OR "Counseling and testing" OR "Counselling and testing" OR HTC OR VCT)) OR CHTC OR CVCT)) ) | Search modes - Boolean/Phrase | 29,074 |
| S29 | S24 OR S25 OR S26 OR S27 OR S28 | Search modes - Boolean/Phrase | 138,312 |
| S30 | (MH "Developing Countries") OR (MH "Africa") OR (MH "Africa South of the Sahara+") OR (MH "Asia") OR (MH "Asia, Central") OR (MH "Kazakhstan") OR (MH "Kyrgyzstan") OR (MH "Tajikistan") OR (MH "Turkmenistan") OR (MH "Uzbekistan") OR (MH "Cambodia") OR (MH "East Timor") OR (MH "Indonesia") OR (MH "Laos") OR (MH "Malaysia") OR (MH "Myanmar") OR (MH "Philippines") OR (MH "Thailand") OR (MH "Timor") OR (MH "Vietnam") OR (MH "Bangladesh") OR (MH "Bhutan") OR (MH "India") OR (MH "Afghanistan") OR (MH "Iraq") OR (MH "Jordan") OR (MH "Lebanon") OR (MH "Syria") OR (MH "Turkey") OR (MH "Yemen") OR (MH "Nepal") OR (MH "Pakistan") OR (MH "Sri Lanka") OR (MH "Mongolia") OR (MH "North Korea") OR (MH "Europe, Eastern") OR (MH "Albania") OR (MH "Bosnia-Herzegovina") OR (MH "Bulgaria") OR (MH "Macedonia (Republic)") OR (MH "Moldova") OR (MH "Romania") OR (MH "Russia") OR (MH "Serbia") OR (MH "Ukraine") OR (MH "Low and Middle Income Countries") OR (MH "Papua New Guinea") OR (MH "Guam") OR (MH "Micronesia") OR (MH "American Samoa") OR (MH "Independent State of Samoa") OR (MH "Samoa") OR (MH "Asia, Southeastern") OR (MH "West Indies") OR (MH "Cuba") OR (MH "Dominica") OR (MH "Dominican Republic") OR (MH "Haiti") OR (MH "Jamaica") OR (MH "Central America") OR (MH "Belize") OR (MH "Costa Rica") OR (MH "El Salvador") OR (MH "Guatemala") OR (MH "Honduras") OR (MH "Nicaragua") OR (MH "Panama") OR (MH "Mexico") OR (MH "South America") OR (MH "Argentina") OR (MH "Bolivia") OR (MH "Brazil") OR (MH "Colombia") OR (MH "Ecuador") OR (MH "Guyana") OR (MH "Paraguay") OR (MH "Peru") OR (MH "Suriname") OR (MH "Venezuela") OR (MH "Africa, Northern+") | Search modes - Boolean/Phrase | 124,074 |
| S31 | TI ( (LMIC OR ((Developing OR Majority OR “Less developed” OR “low resource*” OR disadvantaged OR “resource limited” OR poor OR “low income*” OR “lower income” OR "low and middle income*") N1 (countr* OR region* OR nation? OR area* OR econom*)) OR caribbean* OR “west indi*” OR cuba* OR dominica* OR grenada* OR haiti* OR jamaica* OR “saint lucia*” OR "saint vincent and the grenadines" OR “central america*” OR belize* OR “costa rica*” OR “el salvador*” OR guatemala* OR hondura* OR nicaragua* OR panama* OR “latin america*” OR mexic* OR “south america*” OR argentin* OR bolivia* OR brazil* OR colombia* OR ecuador* OR guyana* OR paraguay* OR peru* OR suriname* OR venezuela* OR asia* OR kazakhstan* OR kyrgyzstan* OR tajikistan* OR turkmenistan* OR uzbekistan* OR russia* OR siberia* OR cambodia* OR “east timor*” OR indonesia* OR lao* OR malaysia* OR myanmar* OR philippin* OR thai* OR vietnam* OR bangladesh* OR bhutan* OR india* OR afghanistan* OR iraq* OR jordan* OR leban* OR syria* OR turkey* OR turkish* OR yemen* OR nepal* OR pakistan* OR “sri lanka*” OR china* OR chinese OR korea* OR mongolia* OR albania* OR “bosnia-herzegovina*” OR bulgaria* OR kosovo* OR macedonia* OR moldov* OR montenegro* OR belarus* OR romania* OR serbia* OR ukraine* OR fiji* OR papua* OR vanuatu* OR micronesia* OR guam* OR palau* OR samoa* OR tonga* OR africa* OR algeria* OR egypt* OR libya* OR morocc* OR tunisia* OR cameroon* OR “central african republic*” OR chad* OR cong* OR “equatorial guinea*” OR gabon* OR burundi* OR djibouti* OR eritrea* OR ethiopia* OR kenya* OR rwanda* OR somalia* OR sudan* OR tanzania* OR uganda* OR angola* OR botswana* OR lesotho* OR malawi* OR mozambique* OR namibia* OR “south africa*” OR swaziland* OR zambia* OR zimbabwe* OR benin* OR “burkina faso*” OR “cape verde*” OR “cote d'ivoire*” OR gambia* OR ghana* OR guinea* OR “guinea-bissau*” OR liberia* OR mali* OR mauritania* OR niger* OR senegal* OR “sierra leone*” OR togo*) ) OR AB ( (LMIC OR ((Developing OR Majority OR “Less developed” OR “low resource*” OR disadvantaged OR “resource limited” OR poor OR “low income*” OR “lower income” OR "low and middle income*") N1 (countr* OR region* OR nation? OR area* OR econom*)) OR caribbean* OR “west indi*” OR cuba* OR dominica* OR grenada* OR haiti* OR jamaica* OR “saint lucia*” OR "saint vincent and the grenadines" OR “central america*” OR belize* OR “costa rica*” OR “el salvador*” OR guatemala* OR hondura* OR nicaragua* OR panama* OR “latin america*” OR mexic* OR “south america*” OR argentin* OR bolivia* OR brazil* OR colombia* OR ecuador* OR guyana* OR paraguay* OR peru* OR suriname* OR venezuela* OR asia* OR kazakhstan* OR kyrgyzstan* OR tajikistan* OR turkmenistan* OR uzbekistan* OR russia* OR siberia* OR cambodia* OR “east timor*” OR indonesia* OR lao* OR malaysia* OR myanmar* OR philippin* OR thai* OR vietnam* OR bangladesh* OR bhutan* OR india* OR afghanistan* OR iraq* OR jordan* OR leban* OR syria* OR turkey* OR turkish* OR yemen* OR nepal* OR pakistan* OR “sri lanka*” OR china* OR chinese OR korea* OR mongolia* OR albania* OR “bosnia-herzegovina*” OR bulgaria* OR kosovo* OR macedonia* OR moldov* OR montenegro* OR belarus* OR romania* OR serbia* OR ukraine* OR fiji* OR papua* OR vanuatu* OR micronesia* OR guam* OR palau* OR samoa* OR tonga* OR africa* OR algeria* OR egypt* OR libya* OR morocc* OR tunisia* OR cameroon* OR “central african republic*” OR chad* OR cong* OR “equatorial guinea*” OR gabon* OR burundi* OR djibouti* OR eritrea* OR ethiopia* OR kenya* OR rwanda* OR somalia* OR sudan* OR tanzania* OR uganda* OR angola* OR botswana* OR lesotho* OR malawi* OR mozambique* OR namibia* OR “south africa*” OR swaziland* OR zambia* OR zimbabwe* OR benin* OR “burkina faso*” OR “cape verde*” OR “cote d'ivoire*” OR gambia* OR ghana* OR guinea* OR “guinea-bissau*” OR liberia* OR mali* OR mauritania* OR niger* OR senegal* OR “sierra leone*” OR togo*) ) | Search modes - Boolean/Phrase | 223,313 |
| S32 | S30 OR S31 | Search modes - Boolean/Phrase | 274,001 |
| S33 | S16 AND S23 AND S29 AND S32 | Search modes - Boolean/Phrase | 107 |
