# Supplementary material for: Improving male involvement in antenatal care in low and middle-income countries to prevent mother to child transmission of HIV: A realist review
Source: PLoS One. 2020 Oct 15;15(10):e0240087. doi: 10.1371/journal.pone.0240087 (PMC7561142; doi:10.1371/journal.pone.0240087)
Supplement: S2 Appendix — (DOCX) [file pone.0240087.s002.docx]

# S2 Appendix – Search Terms and Results as at12/06/20

## S1 Appendix details the search strategy undertaken 12/06/2020. Databases utilised were Ovid MEDLINE, Embase, CINAHL, Cochrane Database of Systematic Reviews, Cochrane Central Register of Controlled Trials, Scopus, Web of Science and ProQuest.

| **Database** | **Citations retrieved (n)** |
| --- | --- |
| Ovid MEDLINE(R) Epub Ahead of Print, In-Process & Other Non-Indexed Citations, Ovid MEDLINE(R) Daily, Ovid MEDLINE and Versions(R) | 323 |
| Embase (Ovid), 1974 to 2017 May 26 | 458 |
| CINAHL (EBSCOhost) | 67 |
| Cochrane Database of Systematic Reviews: Issue 5 of 12, May 2017 | 21 |
| Cochrane Central Register of Controlled Trials: Issue 4 of 12, April 2017 | 75 |
| Scopus | 98 |
| Web of Science | 109 |
| ProQuest (Health & Medicine; Social Sciences subsets) | 115 |
| Total before duplicates removed | 1266 |
| Total after duplicates removed | 741 |

**Database: Ovid MEDLINE(R) and Epub Ahead of Print, In-Process & Other Non-Indexed Citations and Daily <1946 to June 10, 2020>**

**(Ovid)**

1 Pregnancy/ or Pregnant women/ or Prenatal care/ (875264)

2 (Pregnan* or prenatal* or antenatal* or reproductive health).tw,kw. (600890)

3 1 or 2 (1035070)

4 Pregnancy Complications, Infectious/ or Infectious Disease Transmission, Vertical/ (45770)

5 ((("Mother* to child*" or "maternal to child" or vertical*) adj2 transmi*) or PMTCT or MTCT).tw,kw. (13227)

6 4 or 5 (51864)

7 HIV seropositivity/ or HIV-1/ or HIV infections/ (242087)

8 (HIV* or human immunodeficiency virus or PLWHA or PLWHIV or PLHIV).tw,kw. (333632)

9 7 or 8 (361255)

10 (3 or 6) and 9 (27113)

11 Men/ or Male/ or Fathers/ or Family Characteristics/ or Spouses/ or Sex Factors/ or Sexual partners/ or Interpersonal relations/ (8596519)

12 ((Male* or men* or partner* or husband* or spous* or couple*) adj4 (involve* or attend* or participat* or return or accompan* or engagement or object* or opposition* or support*)).tw,kw. (65675)

13 gender*.ti. (45559)

14 11 or 12 or 13 (8633300)

15 Counseling/ or Directive Counseling/ or Patient education as topic/ or Behavior therapy/ or Cognitive Therapy/ or Voluntary Programs/ or Health Education/ or Health Promotion/ or Family Planning Services/ (309141)

16 (counsel* or antenatal care or ANC or (Couple* and ("testing and counsel?ing" or "Counsel?ing and testing" or HTC or VCT)) or CHTC or CVCT).tw,kw. (119865)

17 15 or 16 (398875)

18 Developing countries/ (74526)

19 (LMIC or ((Developing or Majority or Less developed or low resource* or disadvantaged or resource limited or poor or low* income* or "low and middle income*") adj3 (countr* or region* or nation? or area* or econom*))).tw,kw. (107793)

20 Caribbean region/ or west indies/ or cuba/ or dominica/ or dominican republic/ or grenada/ or haiti/ or jamaica/ or saint lucia/ or "saint vincent and the grenadines"/ or americas/ or central america/ or belize/ or costa rica/ or el salvador/ or guatemala/ or honduras/ or nicaragua/ or panama/ or "gulf of mexico"/ or latin america/ or mexico/ or south america/ or argentina/ or bolivia/ or brazil/ or colombia/ or ecuador/ or guyana/ or paraguay/ or peru/ or suriname/ or venezuela/ or asia/ or asia, central/ or kazakhstan/ or kyrgyzstan/ or tajikistan/ or turkmenistan/ or uzbekistan/ or asia, northern/ or russia/ or siberia/ or asia, southeastern/ or cambodia/ or east timor/ or indonesia/ or laos/ or malaysia/ or myanmar/ or philippines/ or thailand/ or vietnam/ or asia, western/ or bangladesh/ or bhutan/ or india/ or afghanistan/ or iraq/ or jordan/ or lebanon/ or syria/ or turkey/ or yemen/ or nepal/ or pakistan/ or sri lanka/ or china/ or "democratic people's republic of korea"/ or mongolia/ or europe, eastern/ or albania/ or bosnia-herzegovina/ or bulgaria/ or kosovo/ or "macedonia (republic)"/ or moldova/ or montenegro/ or "republic of belarus"/ or romania/ or serbia/ or ukraine/ or fiji/ or papua new guinea/ or vanuatu/ or micronesia/ or guam/ or palau/ or samoa/ or american samoa/ or tonga/ or africa/ or africa, northern/ or algeria/ or egypt/ or libya/ or morocco/ or tunisia/ or "africa south of the sahara"/ or africa, central/ or cameroon/ or central african republic/ or chad/ or congo/ or "democratic republic of the congo"/ or equatorial guinea/ or gabon/ or africa, eastern/ or burundi/ or djibouti/ or eritrea/ or ethiopia/ or kenya/ or rwanda/ or somalia/ or sudan/ or tanzania/ or uganda/ or africa, southern/ or angola/ or botswana/ or lesotho/ or malawi/ or mozambique/ or namibia/ or south africa/ or swaziland/ or zambia/ or zimbabwe/ or africa, western/ or benin/ or burkina faso/ or cape verde/ or cote d'ivoire/ or gambia/ or ghana/ or guinea/ or guinea-bissau/ or liberia/ or mali/ or mauritania/ or niger/ or nigeria/ or senegal/ or sierra leone/ or togo/ (1030469)

21 (caribbean* or west indi* or cuba* or dominica* or grenada* or haiti* or jamaica* or saint lucia* or "saint vincent and the grenadines" or central america* or belize* or costa rica* or el salvador* or guatemala* or hondura* or nicaragua* or panama* or latin america* or mexic* or south america* or argentin* or bolivia* or brazil* or colombia* or ecuador* or guyana* or paraguay* or peru* or suriname* or venezuela* or asia* or kazakhstan* or kyrgyzstan* or tajikistan* or turkmenistan* or uzbekistan* or russia* or siberia* or cambodia* or east timor* or indonesia* or lao* or malaysia* or myanmar* or philippin* or thai* or vietnam* or bangladesh* or bhutan* or india* or afghanistan* or iraq* or jordan* or leban* or syria* or turkey* or turkish* or yemen* or nepal* or pakistan* or sri lanka* or china* or chinese or korea* or mongolia* or albania* or bosnia-herzegovina* or bulgaria* or kosovo* or macedonia* or moldov* or montenegro* or belarus* or romania* or serbia* or ukraine* or fiji* or papua* or vanuatu* or micronesia* or guam* or palau* or samoa* or tonga* or africa* or algeria* or egypt* or libya* or morocc* or tunisia* or cameroon* or central african republic* or chad* or cong* or equatorial guinea* or gabon* or burundi* or djibouti* or eritrea* or ethiopia* or kenya* or rwanda* or somalia* or sudan* or tanzania* or uganda* or angola* or botswana* or lesotho* or malawi* or mozambique* or namibia* or south africa* or swaziland* or zambia* or zimbabwe* or benin* or burkina faso* or cape verde* or cote d'ivoire* or gambia* or ghana* or guinea* or guinea-bissau* or liberia* or mali* or mauritania* or niger* or senegal* or sierra leone* or togo*).tw,kw. (2664472)

22 18 or 19 or 20 or 21 (2995098)

23 10 and 14 and 17 and 22 (1450)

24 limit 23 to english language (1408)

25 (201706* or 201707* or 201708* or 201709* or 201710* or 201711* or 201712* or 2018* or 2019* or 202001* or 202002* or 202003* or 202004* or 202005* or 202006*).dt,ez,da. (4724875)

26 24 and 25 (323)

**Database: Embase <1974 to 2020 June 10>**

**(Ovid)**

--------------------------------------------------------------------------------

1 adolescent pregnancy/ or pregnancy/ (592273)

2 pregnant woman/ (78381)

3 prenatal care/ (38726)

4 reproductive health/ (17150)

5 (Pregnan* or prenatal* or antenatal* or reproductive health).tw,kw. (739467)

6 or/1-5 (976312)

7 pregnancy complication/ (60999)

8 vertical transmission/ (14845)

9 ((("Mother* to child*" or "maternal to child" or vertical*) adj2 transmi*) or PMTCT or MTCT).tw,kw. (17005)

10 or/7-9 (83667)

11 human immunodeficiency virus infection/ or human immunodeficiency virus 1 infection/ (287169)

12 (HIV* or human immunodeficiency virus or PLWHA or PLWHIV or PLHIV).tw,kw. (424817)

13 or/11-12 (480030)

14 (6 or 10) and 13 (34331)

15 male/ (8926184)

16 father/ (22770)

17 spouse/ or husband/ (17855)

18 married person/ or married man/ (9945)

19 sex difference/ (364158)

20 ((Male* or men* or partner* or husband* or spous* or couple*) adj4 (involve* or attend* or participat* or return or accompan* or engagement or object* or opposition* or support*)).tw,kw. (89873)

21 gender*.ti. (59044)

22 or/15-21 (9061125)

23 counseling/ or directive counseling/ or e-counseling/ or family counseling/ or patient counseling/ or patient guidance/ (119037)

24 patient education/ (112438)

25 behavior therapy/ (42186)

26 cognitive therapy/ (43274)

27 couple therapy/ (432)

28 health education/ (94563)

29 health promotion/ (96567)

30 family planning/ (34683)

31 (counsel* or antenatal care or ANC or (Couple* and ("testing and counsel?ing" or "Counsel?ing and testing" or HTC or VCT)) or CHTC or CVCT).tw,kw. (175114)

32 or/23-31 (583303)

33 developing country/ (93523)

34 (LMIC or ((Developing or Majority or Less developed or low resource* or disadvantaged or resource limited or poor or low* income* or "low and middle income*") adj3 (countr* or region* or nation? or area* or econom*))).tw,kw. (138292)

35 (caribbean* or west indi* or cuba* or dominica* or grenada* or haiti* or jamaica* or saint lucia* or "saint vincent and the grenadines" or central america* or belize* or costa rica* or el salvador* or guatemala* or hondura* or nicaragua* or panama* or latin america* or mexic* or south america* or argentin* or bolivia* or brazil* or colombia* or ecuador* or guyana* or paraguay* or peru* or suriname* or venezuela* or asia* or kazakhstan* or kyrgyzstan* or tajikistan* or turkmenistan* or uzbekistan* or russia* or siberia* or cambodia* or east timor* or indonesia* or lao* or malaysia* or myanmar* or philippin* or thai* or vietnam* or bangladesh* or bhutan* or india* or afghanistan* or iraq* or jordan* or leban* or syria* or turkey* or turkish* or yemen* or nepal* or pakistan* or sri lanka* or china* or chinese or korea* or mongolia* or albania* or bosnia-herzegovina* or bulgaria* or kosovo* or macedonia* or moldov* or montenegro* or belarus* or romania* or serbia* or ukraine* or fiji* or papua* or vanuatu* or micronesia* or guam* or palau* or samoa* or tonga* or africa* or algeria* or egypt* or libya* or morocc* or tunisia* or cameroon* or central african republic* or chad* or cong* or equatorial guinea* or gabon* or burundi* or djibouti* or eritrea* or ethiopia* or kenya* or rwanda* or somalia* or sudan* or tanzania* or uganda* or angola* or botswana* or lesotho* or malawi* or mozambique* or namibia* or south africa* or swaziland* or zambia* or zimbabwe* or benin* or burkina faso* or cape verde* or cote d'ivoire* or gambia* or ghana* or guinea* or guinea-bissau* or liberia* or mali* or mauritania* or niger* or senegal* or sierra leone* or togo*).tw,kw. (3446570)

36 or/33-35 (3545625)

37 14 and 22 and 32 and 36 (1652)

38 limit 37 to english language (1628)

39 (201706* or 201707* or 201708* or 201709* or 201710* or 201711* or 201712* or 2018* or 2019* or 202001* or 202002* or 202003* or 202004* or 202005* or 202006*).dc. (5455476)

40 38 and 39 (458)

**CINAHL Plus with Full Text**

**(EBSCOhost)**

S1 (MH "Pregnancy") 211,761

S2 (MH "Expectant Mothers") 8,337

S3 (MH "Prenatal Care") 18,085

S4 (MH "Reproductive Health") 7,603

S5 TI ( Pregnan* or prenatal* or or "pre-natal*" OR antenatal* or "ante-natal*" OR "reproductive health" ) OR AB ( Pregnan* or prenatal* or or "pre-natal*" OR antenatal* or "ante-natal*" OR "reproductive health" ) 154,427

S6 S1 OR S2 OR S3 OR S4 OR S5 266,959

S7 (MH "Pregnancy Complications, Infectious") 5,847

S8 (MH "Disease Transmission, Vertical") 6,244

S9 TI ( ((("Mother* to child*" or "mothers to child*" OR "maternal to child" or vertical*) N1 transmi*) or PMTCT or MTCT) ) OR AB ( ((("Mother* to child*" or "mothers to child*" OR "maternal to child" or vertical*) N1 transmi*) or PMTCT or MTCT) ) 3,217

S10 S7 OR S8 OR S9 11,631

S11 (MH "Human Immunodeficiency Virus") OR (MH "HIV-1") 10,209

S12 (MH "HIV Seropositivity") 6,024

S13 TI ( HIV* or "human immunodeficiency virus" or PLWHA or PLWHIV or PLHIV) ) OR AB ( HIV* or "human immunodeficiency virus" or PLWHA or PLWHIV or PLHIV) ) 103,315

S14 S11 OR S12 OR S13 105,613

S15 S6 OR S10 269,467

S16 S14 AND S15 10,534

S17 (MH "Men") OR (MH "Married Men") 6,125

S18 (MH "Spouses") 11,577

S19 (MH "Sexual Partners") 9,364

S20 (MH "Sex Factors") 122,033

S21 TI ( ((Male* or men* or partner* or husband* or spous* or couple*) N3 (involve* or attend* or participat* or return or accompan* or engagement or object* or opposition* or support*)) ) OR AB ( ((Male* or men* or partner* or husband* or spous* or couple*) N3 (involve* or attend* or participat* or return or accompan* or engagement or object* or opposition* or support*)) ) 33,883

S22 TI gender 25,113

S23 S17 OR S18 OR S19 OR S20 OR S21 OR S22 185,138

S24 (MH "Couples Counseling") OR (MH "Counseling") 35,046

S25 (MH "HIV Education") OR (MH "Patient Education") OR (MH "Health Education") 102,501

S26 (MH "Health Promotion") 70,178

S27 (MH "Behavior Therapy") OR (MH "Cognitive Therapy") 32,050

S28 TI ( ((counsel* OR “antenatal care” OR ANC OR (Couple* and ("testing and counseling" OR "testing and counselling" OR "Counseling and testing" OR "Counselling and testing" OR HTC OR VCT)) OR CHTC OR CVCT)) ) OR AB ( ((counsel* OR “antenatal care” OR ANC OR (Couple* and ("testing and counseling" OR "testing and counselling" OR "Counseling and testing" OR "Counselling and testing" OR HTC OR VCT)) OR CHTC OR CVCT)) ) 65,087

S29 S24 OR S25 OR S26 OR S27 OR S28 268,432

S30 (MH "Developing Countries") OR (MH "Africa") OR (MH "Africa South of the Sahara+") OR (MH "Asia") OR (MH "Asia, Central") OR (MH "Kazakhstan") OR (MH "Kyrgyzstan") OR (MH "Tajikistan") OR (MH "Turkmenistan") OR (MH "Uzbekistan") OR (MH "Cambodia") OR (MH "East Timor") OR (MH "Indonesia") OR (MH "Laos") OR (MH "Malaysia") OR (MH "Myanmar") OR (MH "Philippines") OR (MH "Thailand") OR (MH "Timor") OR (MH "Vietnam") OR (MH "Bangladesh") OR (MH "Bhutan") OR (MH "India") OR (MH "Afghanistan") OR (MH "Iraq") OR (MH "Jordan") OR (MH "Lebanon") OR (MH "Syria") OR (MH "Turkey") OR (MH "Yemen") OR (MH "Nepal") OR (MH "Pakistan") OR (MH "Sri Lanka") OR (MH "Mongolia") OR (MH "North Korea") OR (MH "Europe, Eastern") OR (MH "Albania") OR (MH "Bosnia-Herzegovina") OR (MH "Bulgaria") OR (MH "Macedonia (Republic)") OR (MH "Moldova") OR (MH "Romania") OR (MH "Russia") OR (MH "Serbia") OR (MH "Ukraine") OR (MH "Low and Middle Income Countries") OR (MH "Papua New Guinea") OR (MH "Guam") OR (MH "Micronesia") OR (MH "American Samoa") OR (MH "Independent State of Samoa") OR (MH "Samoa") OR (MH "Asia, Southeastern") OR (MH "West Indies") OR (MH "Cuba") OR (MH "Dominica") OR (MH "Dominican Republic") OR (MH "Haiti") OR (MH "Jamaica") OR (MH "Central America") OR (MH "Belize") OR (MH "Costa Rica") OR (MH "El Salvador") OR (MH "Guatemala") OR (MH "Honduras") OR (MH "Nicaragua") OR (MH "Panama") OR (MH "Mexico") OR (MH "South America") OR (MH "Argentina") OR (MH "Bolivia") OR (MH "Brazil") OR (MH "Colombia") OR (MH "Ecuador") OR (MH "Guyana") OR (MH "Paraguay") OR (MH "Peru") OR (MH "Suriname") OR (MH "Venezuela") OR (MH "Africa, Northern+") 309,884

S31 TI ( (LMIC OR ((Developing OR Majority OR “Less developed” OR “low resource*” OR disadvantaged OR “resource limited” OR poor OR “low income*” OR “lower income” OR "low and middle income*") N1 (countr* OR region* OR nation? OR area* OR econom*)) OR caribbean* OR “west indi*” OR cuba* OR dominica* OR grenada* OR haiti* OR jamaica* OR “saint lucia*” OR "saint vincent and the grenadines" OR “central america*” OR belize* OR “costa rica*” OR “el salvador*” OR guatemala* OR hondura* OR nicaragua* OR panama* OR “latin america*” OR mexic* OR “south america*” OR argentin* OR bolivia* OR brazil* OR colombia* OR ecuador* OR guyana* OR paraguay* OR peru* OR suriname* OR venezuela* OR asia* OR kazakhstan* OR kyrgyzstan* OR tajikistan* OR turkmenistan* OR uzbekistan* OR russia* OR siberia* OR cambodia* OR “east timor*” OR indonesia* OR lao* OR malaysia* OR myanmar* OR philippin* OR thai* OR vietnam* OR bangladesh* OR bhutan* OR india* OR afghanistan* OR iraq* OR jordan* OR leban* OR syria* OR turkey* OR turkish* OR yemen* OR nepal* OR pakistan* OR “sri lanka*” OR china* OR chinese OR korea* OR mongolia* OR albania* OR “bosnia-herzegovina*” OR bulgaria* OR kosovo* OR macedonia* OR moldov* OR montenegro* OR belarus* OR romania* OR serbia* OR ukraine* OR fiji* OR papua* OR vanuatu* OR micronesia* OR guam* OR palau* OR samoa* OR tonga* OR africa* OR algeria* OR egypt* OR libya* OR morocc* OR tunisia* OR cameroon* OR “central african republic*” OR chad* OR cong* OR “equatorial guinea*” OR gabon* OR burundi* OR djibouti* OR eritrea* OR ethiopia* OR kenya* OR rwanda* OR somalia* OR sudan* OR tanzania* OR uganda* OR angola* OR botswana* OR lesotho* OR malawi* OR mozambique* OR namibia* OR “south africa*” OR swaziland* OR zambia* OR zimbabwe* OR benin* OR “burkina faso*” OR “cape verde*” OR “cote d'ivoire*” OR gambia* OR ghana* OR guinea* OR “guinea-bissau*” OR liberia* OR mali* OR mauritania* OR niger* OR senegal* OR “sierra leone*” OR togo*) ) OR AB ( (LMIC OR ((Developing OR Majority OR “Less developed” OR “low resource*” OR disadvantaged OR “resource limited” OR poor OR “low income*” OR “lower income” OR "low and middle income*") N1 (countr* OR region* OR nation? OR area* OR econom*)) OR caribbean* OR “west indi*” OR cuba* OR dominica* OR grenada* OR haiti* OR jamaica* OR “saint lucia*” OR "saint vincent and the grenadines" OR “central america*” OR belize* OR “costa rica*” OR “el salvador*” OR guatemala* OR hondura* OR nicaragua* OR panama* OR “latin america*” OR mexic* OR “south america*” OR argentin* OR bolivia* OR brazil* OR colombia* OR ecuador* OR guyana* OR paraguay* OR peru* OR suriname* OR venezuela* OR asia* OR kazakhstan* OR kyrgyzstan* OR tajikistan* OR turkmenistan* OR uzbekistan* OR russia* OR siberia* OR cambodia* OR “east timor*” OR indonesia* OR lao* OR malaysia* OR myanmar* OR philippin* OR thai* OR vietnam* OR bangladesh* OR bhutan* OR india* OR afghanistan* OR iraq* OR jordan* OR leban* OR syria* OR turkey* OR turkish* OR yemen* OR nepal* OR pakistan* OR “sri lanka*” OR china* OR chinese OR korea* OR mongolia* OR albania* OR “bosnia-herzegovina*” OR bulgaria* OR kosovo* OR macedonia* OR moldov* OR montenegro* OR belarus* OR romania* OR serbia* OR ukraine* OR fiji* OR papua* OR vanuatu* OR micronesia* OR guam* OR palau* OR samoa* OR tonga* OR africa* OR algeria* OR egypt* OR libya* OR morocc* OR tunisia* OR cameroon* OR “central african republic*” OR chad* OR cong* OR “equatorial guinea*” OR gabon* OR burundi* OR djibouti* OR eritrea* OR ethiopia* OR kenya* OR rwanda* OR somalia* OR sudan* OR tanzania* OR uganda* OR angola* OR botswana* OR lesotho* OR malawi* OR mozambique* OR namibia* OR “south africa*” OR swaziland* OR zambia* OR zimbabwe* OR benin* OR “burkina faso*” OR “cape verde*” OR “cote d'ivoire*” OR gambia* OR ghana* OR guinea* OR “guinea-bissau*” OR liberia* OR mali* OR mauritania* OR niger* OR senegal* OR “sierra leone*” OR togo*) ) 609,939

S32 S30 OR S31 711,811

S33 S16 AND S23 AND S29 AND S32 318

S34 EM 201706- 1,330,373

S35 S33 AND S34 67

**Cochrane Database of Systematic Reviews: Issue 6 of 12, June 2020; N=21**

**(Wiley)**

(Pregnan* OR prenatal* OR “pre-natal*” OR antenatal* OR “ante-natal*” OR “reproductive health” OR (("Mother to child*" OR “mothers to child*” OR "maternal to child" OR vertical*) NEAR/1 transmi*) OR PMTCT OR MTCT) AND (HIV* OR “human immunodeficiency virus” OR PLWHA OR PLWHIV OR PLHIV) AND (((Male* OR men* OR partner* OR husband* OR spous* OR couple*) NEAR/3 (involve* OR attend* OR participat* OR return OR accompan* OR engagement OR object* OR opposition* OR support*)) OR gender*) AND (counsel* OR “antenatal care” OR ANC OR (Couple* AND ("testing and counseling" OR "testing and counselling" OR "Counseling and testing" OR "Counselling and testing" OR HTC OR VCT)) OR CHTC OR CVCT) AND (LMIC OR ((Developing OR Majority OR “Less developed” OR “low resource*” OR disadvantaged OR “resource limited” OR poor OR “low income*” OR “lower income” OR "low and middle income*") NEAR/2 (countr* OR region* OR nation? OR area* OR econom*)) OR caribbean* OR “west indi*” OR cuba* OR dominica* OR grenada* OR haiti* OR jamaica* OR “saint lucia*” OR "saint vincent and the grenadines" OR “central america*” OR belize* OR “costa rica*” OR “el salvador*” OR guatemala* OR hondura* OR nicaragua* OR panama* OR “latin america*” OR mexic* OR “south america*” OR argentin* OR bolivia* OR brazil* OR colombia* OR ecuador* OR guyana* OR paraguay* OR peru* OR suriname* OR venezuela* OR asia* OR kazakhstan* OR kyrgyzstan* OR tajikistan* OR turkmenistan* OR uzbekistan* OR russia* OR siberia* OR cambodia* OR “east timor*” OR indonesia* OR lao* OR malaysia* OR myanmar* OR philippin* OR thai* OR vietnam* OR bangladesh* OR bhutan* OR india* OR afghanistan* OR iraq* OR jordan* OR leban* OR syria* OR turkey* OR turkish* OR yemen* OR nepal* OR pakistan* OR “sri lanka*” OR china* OR chinese OR korea* OR mongolia* OR albania* OR “bosnia-herzegovina*” OR bulgaria* OR kosovo* OR macedonia* OR moldov* OR montenegro* OR belarus* OR romania* OR serbia* OR ukraine* OR fiji* OR papua* OR vanuatu* OR micronesia* OR guam* OR palau* OR samoa* OR tonga* OR africa* OR algeria* OR egypt* OR libya* OR morocc* OR tunisia* OR cameroon* OR “central african republic*” OR chad* OR cong* OR “equatorial guinea*” OR gabon* OR burundi* OR djibouti* OR eritrea* OR ethiopia* OR kenya* OR rwanda* OR somalia* OR sudan* OR tanzania* OR uganda* OR angola* OR botswana* OR lesotho* OR malawi* OR mozambique* OR namibia* OR “south africa*” OR swaziland* OR zambia* OR zimbabwe* OR benin* OR “burkina faso*” OR “cape verde*” OR “cote d'ivoire*” OR gambia* OR ghana* OR guinea* OR “guinea-bissau*” OR liberia* OR mali* OR mauritania* OR niger* OR senegal* OR “sierra leone*” OR togo*)

Limited with Cochrane Library publication date from Jun 2017 to Jun 2020

**Cochrane Central Register of Controlled Trials: Issue 6 of 12, June 2020; N=75**

**(Wiley)**

(Pregnan* OR prenatal* OR “pre-natal*” OR antenatal* OR “ante-natal*” OR “reproductive health” OR (("Mother to child*" OR “mothers to child*” OR "maternal to child" OR vertical*) NEAR/1 transmi*) OR PMTCT OR MTCT) AND (HIV* OR “human immunodeficiency virus” OR PLWHA OR PLWHIV OR PLHIV) AND (((Male* OR men* OR partner* OR husband* OR spous* OR couple*) NEAR/3 (involve* OR attend* OR participat* OR return OR accompan* OR engagement OR object* OR opposition* OR support*)) OR gender*) AND (counsel* OR “antenatal care” OR ANC OR (Couple* AND ("testing and counseling" OR "testing and counselling" OR "Counseling and testing" OR "Counselling and testing" OR HTC OR VCT)) OR CHTC OR CVCT) AND (LMIC OR ((Developing OR Majority OR “Less developed” OR “low resource*” OR disadvantaged OR “resource limited” OR poor OR “low income*” OR “lower income” OR "low and middle income*") NEAR/2 (countr* OR region* OR nation? OR area* OR econom*)) OR caribbean* OR “west indi*” OR cuba* OR dominica* OR grenada* OR haiti* OR jamaica* OR “saint lucia*” OR "saint vincent and the grenadines" OR “central america*” OR belize* OR “costa rica*” OR “el salvador*” OR guatemala* OR hondura* OR nicaragua* OR panama* OR “latin america*” OR mexic* OR “south america*” OR argentin* OR bolivia* OR brazil* OR colombia* OR ecuador* OR guyana* OR paraguay* OR peru* OR suriname* OR venezuela* OR asia* OR kazakhstan* OR kyrgyzstan* OR tajikistan* OR turkmenistan* OR uzbekistan* OR russia* OR siberia* OR cambodia* OR “east timor*” OR indonesia* OR lao* OR malaysia* OR myanmar* OR philippin* OR thai* OR vietnam* OR bangladesh* OR bhutan* OR india* OR afghanistan* OR iraq* OR jordan* OR leban* OR syria* OR turkey* OR turkish* OR yemen* OR nepal* OR pakistan* OR “sri lanka*” OR china* OR chinese OR korea* OR mongolia* OR albania* OR “bosnia-herzegovina*” OR bulgaria* OR kosovo* OR macedonia* OR moldov* OR montenegro* OR belarus* OR romania* OR serbia* OR ukraine* OR fiji* OR papua* OR vanuatu* OR micronesia* OR guam* OR palau* OR samoa* OR tonga* OR africa* OR algeria* OR egypt* OR libya* OR morocc* OR tunisia* OR cameroon* OR “central african republic*” OR chad* OR cong* OR “equatorial guinea*” OR gabon* OR burundi* OR djibouti* OR eritrea* OR ethiopia* OR kenya* OR rwanda* OR somalia* OR sudan* OR tanzania* OR uganda* OR angola* OR botswana* OR lesotho* OR malawi* OR mozambique* OR namibia* OR “south africa*” OR swaziland* OR zambia* OR zimbabwe* OR benin* OR “burkina faso*” OR “cape verde*” OR “cote d'ivoire*” OR gambia* OR ghana* OR guinea* OR “guinea-bissau*” OR liberia* OR mali* OR mauritania* OR niger* OR senegal* OR “sierra leone*” OR togo*)

Limited with Cochrane Library publication date from Jun 2017 to Jun 2020

**Scopus; N=98**

( TITLE-ABS-KEY ( ( pregnan* OR prenatal* OR "pre-natal*" OR antenatal* OR "ante-natal*" OR "reproductive health" OR ( ( "Mother to child*" OR "mothers to child*" OR "maternal to child" OR vertical* ) W/1 transmi* ) OR pmtct OR mtct ) AND ( hiv* OR "human immunodeficiency virus" OR plwha OR plwhiv OR plhiv ) ) ) AND ( TITLE-ABS-KEY ( ( ( male* OR men* OR partner* OR husband* OR spous* OR couple* ) W/3 ( involve* OR attend* OR participat* OR return OR accompan* OR engagement OR object* OR opposition* OR support* ) ) ) OR TITLE ( gender* ) ) AND ( TITLE-ABS-KEY ( ( counsel* OR "antenatal care" OR anc OR ( couple* AND ( "testing and counsel*ing" OR "Counsel*ing and testing" OR htc OR vct ) ) OR chtc OR cvct ) AND ( lmic OR ( ( developing OR majority OR "Less developed" OR "low resource*" OR disadvantaged OR "resource limited" OR poor OR "low income*" OR "lower income" OR "low and middle income*" ) W/2 ( countr* OR region* OR nation? OR area* OR econom* ) ) OR caribbean* OR "west indi*" OR cuba* OR dominica* OR grenada* OR haiti* OR jamaica* OR "saint lucia*" OR "saint vincent and the grenadines" OR "central america*" OR belize* OR "costa rica*" OR "el salvador*" OR guatemala* OR hondura* OR nicaragua* OR panama* OR "latin america*" OR mexic* OR "south america*" OR argentin* OR bolivia* OR brazil* OR colombia* OR ecuador* OR guyana* OR paraguay* OR peru* OR suriname* OR venezuela* OR asia* OR kazakhstan* OR kyrgyzstan* OR tajikistan* OR turkmenistan* OR uzbekistan* OR russia* OR siberia* OR cambodia* OR "east timor*" OR indonesia* OR lao* OR malaysia* OR myanmar* OR philippin* OR thai* OR vietnam* OR bangladesh* OR bhutan* OR india* OR afghanistan* OR iraq* OR jordan* OR leban* OR syria* OR turkey* OR turkish* OR yemen* OR nepal* OR pakistan* OR "sri lanka*" OR china* OR chinese OR korea* OR mongolia* OR albania* OR "bosnia-herzegovina*" OR bulgaria* OR kosovo* OR macedonia* OR moldov* OR montenegro* OR belarus* OR romania* OR serbia* OR ukraine* OR fiji* OR papua* OR vanuatu* OR micronesia* OR guam* OR palau* OR samoa* OR tonga* OR africa* OR algeria* OR egypt* OR libya* OR morocc* OR tunisia* OR cameroon* OR "central african republic*" OR chad* OR cong* OR "equatorial guinea*" OR gabon* OR burundi* OR djibouti* OR eritrea* OR ethiopia* OR kenya* OR rwanda* OR somalia* OR sudan* OR tanzania* OR uganda* OR angola* OR botswana* OR lesotho* OR malawi* OR mozambique* OR namibia* OR "south africa*" OR swaziland* OR zambia* OR zimbabwe* OR benin* OR "burkina faso*" OR "cape verde*" OR "cote d'ivoire*" OR gambia* OR ghana* OR guinea* OR "guinea-bissau*" OR liberia* OR mali* OR mauritania* OR niger* OR senegal* OR "sierra leone*" OR togo* ) ) ) AND ORIG-LOAD-DATE > 20170601 AND ( LIMIT-TO ( LANGUAGE , "English" ) )

**Web of Science; N=109**


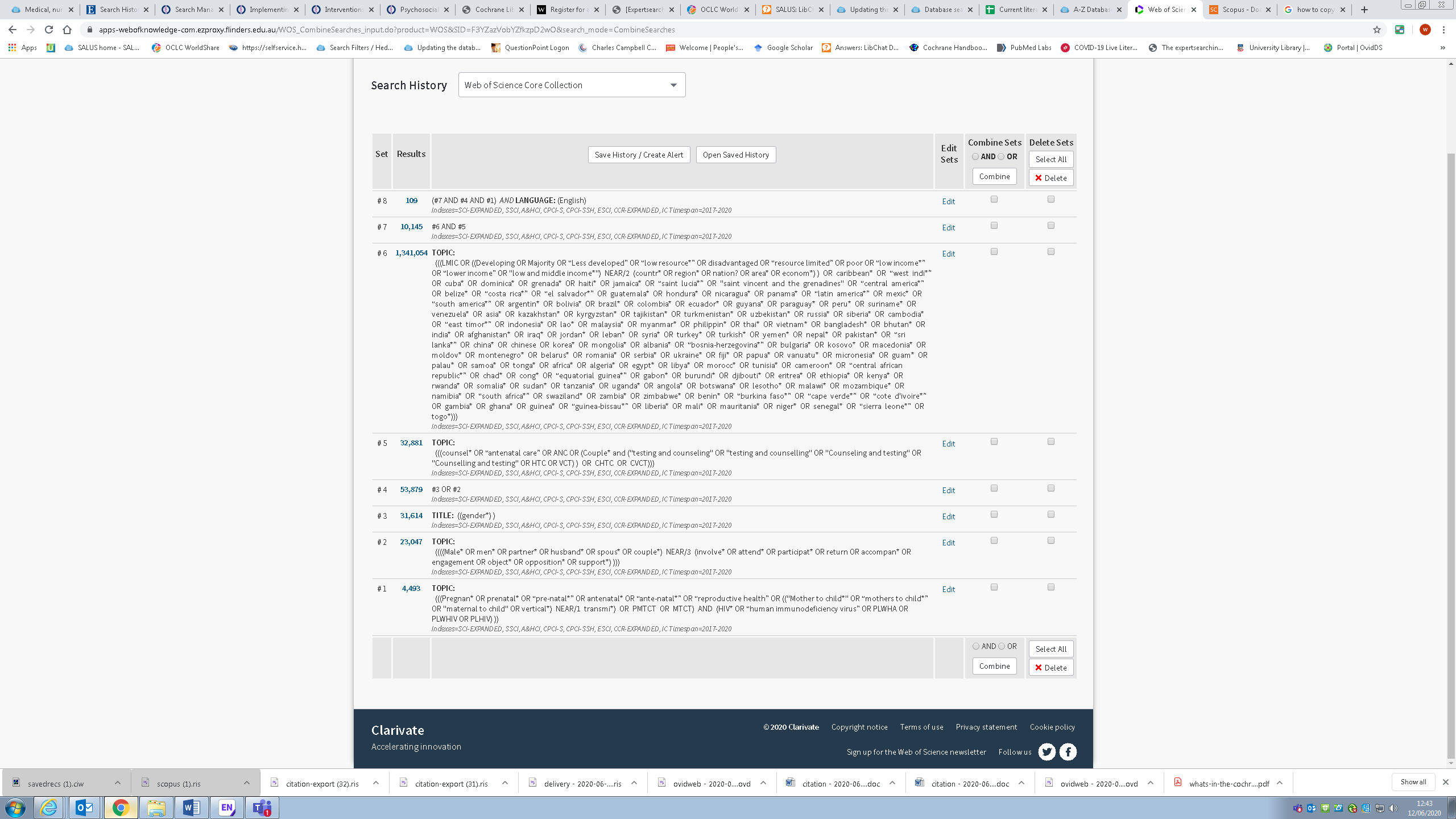


**Proquest; N=115**

all((Pregnan* OR prenatal* OR "pre-natal*" OR antenatal* OR "ante-natal*" OR "reproductive health" OR (("Mother to child*" OR "mothers to child*" OR "maternal to child" OR vertical*) NEAR/1 transmi*) OR PMTCT OR MTCT) AND (HIV* OR "human immunodeficiency virus" OR PLWHA OR PLWHIV OR PLHIV) AND (((Male* OR men* OR partner* OR husband* OR spous* OR couple*) NEAR/3 (involve* OR attend* OR participat* OR return OR accompan* OR engagement OR object* OR opposition* OR support*)) OR gender*) AND (counsel* OR "antenatal care" OR ANC OR (Couple* AND ("testing and counseling" OR "testing and counselling" OR "Counseling and testing" OR "Counselling and testing" OR HTC OR VCT)) OR CHTC OR CVCT) AND (LMIC OR ((Developing OR Majority OR "Less developed" OR "low resource*" OR disadvantaged OR "resource limited" OR poor OR "low income*" OR "lower income" OR "low and middle income*") NEAR/2 (countr* OR region* OR nation? OR area* OR econom*)) OR caribbean* OR "west indi*" OR cuba* OR dominica* OR grenada* OR haiti* OR jamaica* OR "saint lucia*" OR "saint vincent and the grenadines" OR "central america*" OR belize* OR "costa rica*" OR "el salvador*" OR guatemala* OR hondura* OR nicaragua* OR panama* OR "latin america*" OR mexic* OR "south america*" OR argentin* OR bolivia* OR brazil* OR colombia* OR ecuador* OR guyana* OR paraguay* OR peru* OR suriname* OR venezuela* OR asia* OR kazakhstan* OR kyrgyzstan* OR tajikistan* OR turkmenistan* OR uzbekistan* OR russia* OR siberia* OR cambodia* OR "east timor*" OR indonesia* OR lao* OR malaysia* OR myanmar* OR philippin* OR thai* OR vietnam* OR bangladesh* OR bhutan* OR india* OR afghanistan* OR iraq* OR jordan* OR leban* OR syria* OR turkey* OR turkish* OR yemen* OR nepal* OR pakistan* OR "sri lanka*" OR china* OR chinese OR korea* OR mongolia* OR albania* OR "bosnia-herzegovina*" OR bulgaria* OR kosovo* OR macedonia* OR moldov* OR montenegro* OR belarus* OR romania* OR serbia* OR ukraine* OR fiji* OR papua* OR vanuatu* OR micronesia* OR guam* OR palau* OR samoa* OR tonga* OR africa* OR algeria* OR egypt* OR libya* OR morocc* OR tunisia* OR cameroon* OR "central african republic*" OR chad* OR cong* OR "equatorial guinea*" OR gabon* OR burundi* OR djibouti* OR eritrea* OR ethiopia* OR kenya* OR rwanda* OR somalia* OR sudan* OR tanzania* OR uganda* OR angola* OR botswana* OR lesotho* OR malawi* OR mozambique* OR namibia* OR "south africa*" OR swaziland* OR zambia* OR zimbabwe* OR benin* OR "burkina faso*" OR "cape verde*" OR "cote d'ivoire*" OR gambia* OR ghana* OR guinea* OR "guinea-bissau*" OR liberia* OR mali* OR mauritania* OR niger* OR senegal* OR "sierra leone*" OR togo*))

Additional limits - Date: After 01 June 2017
